# Emotion Dysregulation as a Core Feature of Borderline Personality Disorder: Associations with Impulsivity and Symptom Severity in Emerging Adulthood

**DOI:** 10.3390/jcm15031047

**Published:** 2026-01-28

**Authors:** Anaïs Mungo, Marie Delhaye, Matthieu Hein

**Affiliations:** 1 Department of Psychiatry, CH Le Domaine-ULB, Université Libre de Bruxelles (ULB), 1420 Braine l’Alleud, Belgium; anais.mungo@ulb.be; 2 Unit 2, Department of Psychiatry, La Ramée, Epsylon, 1180 Brussels, Belgium; marie.delhaye@umons.ac.be; 3 Laboratoire de Psychologie Médicale et Addictologie (ULB312), CHU Brugmann, Université Libre de Bruxelles (ULB), 1020 Brussels, Belgium

**Keywords:** borderline personality disorder, emotion dysregulation, young adults, impulsivity, DERS (Difficulties in Emotion Regulation Scale), emotional impulsivity, early intervention

## Abstract

**Objectives:** This study aimed to explore the relationship between emotion dysregulation (ED), impulsivity, and symptom severity in emerging adults (16–25 years) diagnosed with Borderline Personality Disorder (BPD). Specifically, it sought to determine which ED dimensions differentiate BPD from non-clinical, independently of anxiety and depression, and how these relate to clinical features of BPD. **Methods:** A total of 184 participants (BPD = 44, non-clinical group = 140) completed standardized assessments, including the DERS (ED), UPPS-P (impulsivity), DIB-R (BPD), BDI-II (depression), and STAI-T (trait anxiety). Analyses included Mann–Whitney tests, quantile and logistic regressions, and Spearman correlations, adjusting for clinical covariates. **Results:** BPD participants scored significantly higher on all DERS subscales (*p* < 0.001). Adjusted regressions identified Impulse, Awareness, and Clarity as key discriminators (ORs: 5.91, 3.56, 2.90), and a total DERS score >129 increased BPD likelihood twelvefold. ED dimensions were associated with DIB-R symptom severity, especially Impulse and Strategies. Only Clarity showed a negative correlation with suicide attempts, suggesting greater emotional confusion was linked to fewer reported attempts. ED also correlated with urgency traits on the UPPS-P. **Conclusions:** ED—particularly emotional impulsivity, poor awareness, and low clarity—emerges as a core marker of BPD in emerging adulthood. These findings underscore the importance of early intervention strategies targeting emotional identification, modulation, and impulsivity control to mitigate clinical severity and long-term risk.

## 1. Introduction

### 1.1. Developmental Transition and Emotional Vulnerability

Adolescence and early adulthood (ages 16–25) represent a crucial phase of development, characterized by major biological, cognitive, affective, and social transformations. This transitional period is also when vulnerability to mental disorders is at its highest: more than 60% of psychiatric disorders emerge before the age of 25, with a peak incidence around 14.5 years [1]. This developmental stage is particularly sensitive due to the interaction between early environmental factors (e.g., trauma, family dysfunction) and ongoing neurobiological maturation. Contemporary neurodevelopmental models emphasize that emotion regulation relies on the progressive and hierarchical maturation of interconnected fronto-limbic circuits rather than on isolated brain regions. During adolescence and emerging adulthood, subcortical systems involved in emotional reactivity and reward processing mature earlier, whereas prefrontal and cortico-cortical networks supporting cognitive control, emotional regulation, and behavioral inhibition continue to develop in a more protracted manner. This asynchronous, circuit-based maturation results in a transient imbalance in functional connectivity, characterized by heightened emotional reactivity and still-limited regulatory control. These developmental dynamics are further modulated by pubertal and age-related changes in dopaminergic systems, increasing sensitivity to emotionally and socially salient stimuli and contributing to a heightened vulnerability to emotionally driven impulsive behaviors [2,3].

### 1.2. Emotional Dysregulation as a Psychopathological Risk Factor

In this context, emotional dysregulation—defined as the difficulty in identifying, understanding, accepting, and managing intense emotions—is particularly salient during adolescence [4]. While a certain degree of emotional instability is expected during development, it can become a major psychopathological risk factor when accompanied by early trauma or invalidating environments [1,5]. Recent research confirms that early relational experiences marked by insecure attachment or affective trauma promote the development of maladaptive emotion regulation strategies, such as avoidance or dissociation. These responses reinforce rigid emotional patterns, contributing to increased vulnerability to borderline traits, particularly those related to affective instability and self-damaging behaviors [6,7]. This fragility lies at the core of borderline personality disorder (BPD), whose early onset and symptomatic expression are strongly linked to impulsivity and emotional dysregulation.

Moreover, emotional dysregulation in BPD is not limited to affective hyperreactivity. It can also manifest as inhibition or emotional numbing. Van Dijke and Ford (2015) [8] demonstrated that these two dimensions—emotional under-regulation and over-regulation—are rooted in distinct attachment strategies: the former hyperactivated, characterized by fear of abandonment, and the latter deactivated, associated with fear of closeness. These profiles highlight the complexity of emotional experience in BPD and support the hypothesis of bidimensional dysregulation [8].

The transition from adolescence to early adulthood may therefore be conceptualized as a developmentally sensitive period for emotion regulation. During this phase, ongoing maturation of socio-affective and regulatory processes coincides with increased exposure to relational, academic, and autonomy-related challenges, while neuro-affective systems remain particularly reactive. Under conditions of early trauma or invalidating environments, these developmental vulnerabilities may consolidate into persistent patterns of emotional dysregulation, thereby increasing the risk for the emergence of personality disorders, particularly BPD [9,10].

### 1.3. The Biosocial Model and Developmental Trajectories of BPD

Recent studies confirm that childhood trauma, particularly experiences of abuse and neglect, is significantly associated with the emergence of borderline traits in adulthood. Among the explanatory frameworks proposed, Linehan’s biosocial model (1993) remains the major reference [11]. It posits that emotional dysregulation results from a dynamic interaction between biological vulnerabilities—such as heightened emotional sensitivity, marked impulsivity, and slow emotional recovery—and an invalidating environment in which the child’s emotional responses are minimized, punished, or ignored [11,12].

From this perspective, early interpersonal experiences play a crucial role in shaping emotion regulation capacities and, consequently, in the development of borderline features [13,14]. Longitudinal data from adolescent cohorts support this model: parental invalidation, emotional vulnerability, and impulsivity each contribute independently to emotional dysregulation, which in turn predicts the later severity of borderline symptoms [15].

Extending this conceptual framework, Chapman (2019) emphasizes the reciprocal transaction between constitutional impulsivity and an invalidating environment: a biologically reactive child tends to elicit inadequate parental responses, which progressively reinforce emotional regulation difficulties [16]. This developmental perspective thus sheds light on the evolving trajectory linking impulsive temperament to the clinical manifestations of borderline pathology [16].

From a nosographic standpoint, BPD, as defined by the DSM-V-TR (2022), is characterized by chronic emotional instability, marked impulsivity, identity disturbance, and chaotic interpersonal relationships [17]. BPD is a severe mental disorder affecting approximately 0.7% to 2.7% of adults in the general population [18]. Among adolescents and young adults, community prevalence rates have been estimated at 1–3% [10]. In clinical settings, substantially higher rates have been reported, ranging from 11% to 22% in out-patient psychiatric services and from 22% to 49% among psychiatric inpatients, according to epidemiological studies summarized in the literature [10,18].

### 1.4. Transdiagnostic Dimension of Emotional Dysregulation

Several recent studies support the hypothesis that emotional dysregulation represents a transdiagnostic dimension shared across various disorders characterized by high impulsivity, such as attention-deficit/hyperactivity disorder (ADHD) and BPD.

This transdiagnostic dimension has been particularly well illustrated in the literature comparing BPD and ADHD. Convergently, Bozzatello et al. (2021) emphasize that emotional dysregulation and impulsivity are not specific to BPD but rather constitute transdiagnostic vulnerabilities shared with other emotional and anxiety disorders [19].

ADHD and BPD share common neurodevelopmental vulnerabilities, particularly regarding emotional reactivity, impulsivity, and exposure to early adverse experiences. However, the forms of impulsivity differ: motor and persistent in ADHD, emotional and contextual in BPD [20].

These disorders exhibit similar emotional profiles at both behavioral and neurobiological levels, including heightened emotional reactivity, affective instability, and alterations in the fronto-limbic networks involved in emotion regulation [21,22]. Recent studies using experience sampling methodology (ESM) have shown that adults with BPD and those with ADHD display comparable levels of emotional intensity and instability. However, the triggering contexts of these emotional fluctuations differ: individuals with BPD tend to react more strongly to interpersonal situations, whereas those with ADHD are affected by a broader range of factors, suggesting distinct profiles of emotional reactivity despite overlapping dynamic symptomatology [21].

### 1.5. The DERS as an Assessment Tool and Specificities in BPD

To assess emotional dysregulation, Gratz and Roemer (2004) developed the Difficulties in Emotion Regulation Scale (DERS), which provides a multidimensional evaluation of emotion regulation [23]. The instrument includes six subscales: Awareness (emotional awareness), Clarity (emotional understanding), Nonacceptance (non-acceptance of emotions), Impulse (difficulty controlling behavior under distress), Goals (difficulty pursuing goal-directed behavior when distressed), and Strategies (perceived access to effective regulation strategies) [23]. Recent findings suggest that certain subscales—particularly Impulse and Strategies—may be more sensitive to clinical change following mindfulness-based or DBT-centered interventions, supporting their usefulness in evaluating therapeutic mechanisms of change [24].

The specificity of emotional dysregulation in BPD has been widely documented. In a comparative study, Ibraheim et al. (2017) showed that hospitalized adolescents with BPD scored significantly higher on the DERS, particularly on the Impulse and Strategies dimensions, even after controlling for age, sex, and overall psychiatric severity [4]. Other studies conducted in mixed clinical populations (BPD, complex PTSD, dissociative disorders) have confirmed the relevance of the DERS in differentiating these profiles: individuals with BPD are characterized by a combination of emotional intensity, limited regulation strategies, and distress-related impulsivity [6,22].

These findings confirm that while certain components of emotion regulation are transdiagnostic, they are particularly pronounced in adolescent BPD.

### 1.6. Clinical Implications of Emotional Dysregulation and Emotional Impulsivity

Furthermore, these specific dimensions of emotional dysregulation have major clinical implications. Impulsivity, in particular, is frequently observed in BPD and manifests through behaviors such as self-harm, suicide attempts, risky sexual behavior, or substance abuse [25,26]. This impulsivity is not independent—it often emerges as a mode of expression of acute emotional distress, functioning as a dysfunctional regulation strategy in response to affective overload. Borderline patients tend to rely more on ineffective strategies such as rumination, avoidance, or suppression [27], which in turn heightens emotional reactivity. Sharp et al. (2015) demonstrated that affective hyperactivation and regulatory deficits interfere with mentalization capacity, leading to impulsive reactions in interpersonal contexts [9].

Recent integrative models conceptualize impulsivity not merely as a deficit in inhibitory control but as a transient emotional regulation strategy aimed at escaping or soothing intense affective states. This immediate relief function is thought to be mediated by low distress tolerance, which limits access to more adaptive emotion regulation strategies [28].

Certain DERS subscales—particularly Impulse, Goals, and Strategies—strongly discriminate adolescents with high levels of borderline traits [29]. These dimensions, respectively, reflect difficulties in inhibiting behavior under emotional distress, maintaining goals despite affective intensity, and perceiving oneself as lacking effective emotional management strategies. Waite et al. (2024) recently showed that both positive and negative emotional dysregulation mediate the association between impulsivity and BPD, further emphasizing the relevance of these dimensions for understanding the underlying mechanisms of the disorder [30].

Mortensen et al. (2010) found that women with BPD exhibit significantly higher levels of trait impulsivity, particularly in the motor and non-planning dimensions of the BIS-11 [31]. The authors highlighted that this subjectively perceived impulsivity manifests primarily in emotional contexts, supporting the notion that emotional impulsivity is a central component of borderline functioning. This form of impulsivity, characterized by the tendency to act under the influence of intense negative emotions, reinforces the hypothesis that emotional impulsivity constitutes a key marker of borderline functioning [31].

Cackowski et al. (2014) demonstrated that exposure to acute stress significantly increases behavioral impulsivity—measured by impaired inhibitory control in a Go/No-Go task—among patients with BPD [32]. This stress-induced impulsive reactivity reflects the interaction between motor disinhibition and emotional components of impulsivity, consistent with models of emotional dysregulation in BPD [32].

Finally, particular attention should be given to the links between DERS subdimensions and the various facets of clinical impulsivity. Far from being a unitary construct, impulsivity in BPD encompasses diverse behaviors—self-harm, risky sexual activity, substance use, eating disorders, and suicide attempts—each potentially associated with specific emotional deficits [30].

In a study of adolescents with borderline traits, Fossati et al. (2013) found that the Impulse and Strategies subscales of the DERS significantly predicted the severity of borderline traits, independently of trait impulsivity [29]. These findings underscore the importance of linking emotional dysregulation profiles to different expressions of impulsivity. They converge with those of Moukhtarian et al. (2021), who identified comparable emotional dynamics between ADHD and BPD, thereby illustrating the transdiagnostic dimension of emotional impulsivity in disorders of impulse control [21].

### 1.7. Emotional Dysregulation and Suicidal Behaviors

More than 75% of individuals with BPD report at least one suicide attempt [18], a rate that also applies to adolescents and young adults [9]. These behaviors, far from being solely attributable to depressive states, can be understood as maladaptive attempts to modulate or escape from overwhelming emotional distress [33]. A longitudinal study by Williams et al. (2023) confirmed that the presence of a personality disorder—whether diagnosed categorically or dimensionally—constitutes an independent and cumulative risk factor for suicidal behavior in adolescents, even in the absence of BPD [34]. This study highlights the importance of considering personality pathology as a whole to better understand the chronicity and lethality of suicidal behaviors [34].

Recent evidence indicates that experiential avoidance is a prominent process in BPD, particularly among individuals with greater clinical severity and persistent symptomatology. Given its close association with emotional distress, anxiety, and functional impairment, experiential avoidance may be especially relevant in clinical profiles characterized by elevated suicidal risk [35]. Recent longitudinal data indicate that the severity of suicide attempts among individuals with BPD is more strongly predicted by emotional dysregulation and insecure attachment than by depressive symptoms, confirming the mediating role of emotional dysregulation (as measured by the DERS) between affective vulnerability and suicidal action [7].

In this regard, Williams et al. (2023) showed that the co-occurrence of major depressive disorder (MDD) and personality pathology, particularly borderline traits, significantly increases the frequency, recurrence, and lethality of suicide attempts among adolescents [34]. The MDD + BPD combination amplifies emotional distress and reduces affect regulation capacities, constituting a particularly concerning clinical risk profile [34].

These findings reinforce the notion that emotional dysregulation plays a transdiagnostic role, yet one that is particularly critical in BPD. It emerges as a mediator between affective states and impulsive behavior, making it a key therapeutic target. Early interventions focused on emotion regulation—such as stepped-care approaches and dialectical behavior therapy (DBT) adapted for young adults—have demonstrated significant improvements in regulation strategies and reductions in both suicidal and impulsive behaviors [24,36].

### 1.8. Objectives

In line with early detection recommendations, this study focuses on individuals aged 16–25 years and employs standardized instruments to characterize emotional dysregulation profiles associated with BPD [19].

The present study extends our previous work on the multidimensional profile of impulsivity in young adults with BPD [37]. Here, we explore the complementary dimension of emotion regulation, assessed using the Difficulties in Emotion Regulation Scale (DERS), to deepen the understanding of the emotional processes underlying borderline functioning during the transition to adulthood.

This study examines the relationships between various facets of emotion regulation, clinical impulsivity, and symptom severity in young adults aged 16–25—a developmental phase particularly vulnerable to the onset of psychopathological disorders. This period, characterized by the still-maturing systems of emotional regulation and inhibitory control, provides an ideal framework for investigating the transdiagnostic mechanisms underlying impulsive and self-damaging behaviors.

More specifically, we compare the levels of emotional dysregulation between participants with BPD and a non-clinical group across the six subdimensions of the DERS. We aim to identify which dimensions of emotion regulation are specifically associated with BPD, independently of anxiety and depression levels.

We also examine the relationships between the different DERS subscales and the clinical dimensions of BPD assessed with the Diagnostic Interview for Borderlines—Revised (DIB-R), in order to better understand the emotional profiles associated with symptom severity.

Furthermore, we analyze the association between specific components of emotion regulation and the presence of suicidal behaviors and substance use among participants with BPD, with a specific focus on the link between the number of suicide attempts and particular emotional dimensions.

Finally, we evaluate the relationship between different facets of emotional dysregulation and impulsivity traits measured by the UPPS-P model, focusing particularly on Positive Urgency, Negative Urgency, Lack of Premeditation, and Lack of Perseverance, in order to better characterize the impulsivity profile associated with emotional dysregulation in BPD.

## 2. Materials and Methods

The present study is based on the same clinical cohort as our previously published work examining the multidimensional impulsivity profile in young adults with BPD using the UPPS-P model [37].

The non-clinical sample was recruited from similar sources but was defined using fewer exclusion criteria in the present study. As a result, the non-clinical samples included in the two studies are not strictly identical.

Consequently, some sociodemographic and clinical variables, as well as impulsivity measures assessed with the UPPS-P, partially overlap with those reported in the previous publication. In the present manuscript, these overlapping variables are used for descriptive purposes or as secondary correlates.

Importantly, the primary objective of the current study is distinct and focuses on emotion dysregulation assessed by the Difficulties in Emotion Regulation Scale (DERS), which was not the main outcome in the previous article. No primary analyses or interpretations are duplicated, and the two manuscripts address complementary and non-redundant research questions.

### 2.1. Population

Participants were drawn from both clinical and non-clinical settings. The clinical sample was recruited between January 2021 and October 2023 from inpatient and outpatient services within adolescent and adult psychiatry departments of a general university hospital in the Brussels metropolitan area.

Eligibility for the clinical group required a diagnosis of BPD in accordance with DSM-5 criteria. Diagnoses were established by the treating psychiatrist and independently confirmed by a trained clinical researcher using the semi-structured Diagnostic Interview for Borderlines—Revised (DIB-R). Additional inclusion criteria were age between 16 and 25 years and adequate proficiency in spoken and written French. Exclusion criteria comprised refusal to participate, inability to complete the assessment battery, intellectual disability, lifetime or current psychotic disorder, autism spectrum disorder, and severe somatic conditions (such as cancer, advanced cardiac or renal disease, or progressive neurological disorders) likely to affect short-term prognosis.

Participants with BPD were enrolled irrespective of symptom severity, treatment phase, or care setting (inpatient versus outpatient). No exclusion was applied based on psychiatric comorbidities or acute clinical status. Inclusion was contingent solely on diagnostic confirmation and the capacity to complete self-report questionnaires. Consequently, the clinical sample reflects the clinical heterogeneity and variability typically encountered in routine psychiatric practice.

The non-clinical comparison group was recruited between 2021 and 2023 through multiple sources to ensure diversity and representativeness. Recruitment took place in secondary schools located in Wallonia and in both northern and southern areas of Brussels, as well as among students from the Faculty of Medicine and other faculties of the Université libre de Bruxelles (ULB). Additional volunteers were recruited via informational posters displayed within the university hospital. Inclusion criteria for the non-clinical group were age between 16 and 25 years and sufficient proficiency in French. Exclusion criteria included refusal to participate, inability to complete questionnaires, presence of a current psychiatric disorder, ongoing psychiatric care, current psychotropic medication use, or severe somatic illness as defined above.

In the BPD group, 60 individuals were initially contacted. Of these, 55 met eligibility criteria and consented to participate; complete data were available for 44 participants due to attrition or incomplete assessments. In the non-clinical group, 161 individuals were recruited, with complete datasets obtained for 159 participants. Following exclusion of individuals presenting with a current psychiatric disorder, ongoing psychiatric follow-up, or psychotropic medication use, 140 participants were retained in the non-clinical group.

The final sample comprised 184 participants, including 44 individuals with BPD and 140 non-clinical participants.

### 2.2. Method

All participants were fully informed about the study procedures, and written informed consent was obtained prior to enrollment. For participants under the age of 18, additional written consent was provided by a parent or legal guardian. All assessment instruments were administered in French.

The clinical group included only participants who fulfilled DSM-5 diagnostic criteria for BPD. Diagnostic status was established during a prior clinical evaluation and confirmed using the semi-structured Diagnostic Interview for Borderlines—Revised (DIB-R).

The DIB-R is a semi-structured clinical interview designed to assess the core domains of BPD, namely affective instability, cognitive disturbances, impulsive behaviors, and interpersonal functioning. Items are rated on a three-point scale ranging from 0 (absent) to 2 (clearly present), with an intermediate score of 1 indicating probable presence. Domain scores are aggregated and converted into standardized dimensional indices according to a predefined scoring algorithm, yielding a total score ranging from 0 to 10. Suicide attempts were assessed using clinician-rated information from the DIB-R. Although suicide attempts are embedded within the impulsivity dimension of the DIB-R, the number of lifetime suicide attempts was also extracted and analyzed as a separate clinical variable. The DIB-R has demonstrated robust psychometric properties, including high test–retest reliability, strong inter-rater agreement, and longitudinal stability of borderline symptomatology, with kappa coefficients exceeding 0.75 across domains [38]. A French version of the instrument has been validated, showing satisfactory reliability for both total and dimensional scores in a French-speaking clinical population [39].

Depressive symptom severity was evaluated using the Beck Depression Inventory—Second Edition (BDI-II). This self-report measure comprises 21 items rated on a four-point Likert scale from 0 (absence of symptoms) to 3 (severe symptom expression), resulting in total scores ranging from 0 to 63, with higher scores indicating greater depressive severity. The BDI-II assesses a broad range of depressive manifestations, including cognitive, affective, and somatic symptoms such as sadness, pessimism, self-criticism, fatigue, and appetite or sleep disturbances. Owing to its strong psychometric properties, the BDI-II is extensively used in both clinical and research contexts. Conventional severity thresholds are defined as follows: 0–13 indicating minimal or no depression, 14–19 mild depression, 20–28 moderate depression, and 29–63 severe depression [40].

Trait anxiety was assessed using the Trait subscale of the State-Trait Anxiety Inventory (STAI-T), originally developed by Spielberger et al. [41]. The STAI is a validated self-report instrument composed of two distinct 20-item scales: the State Anxiety Scale (STAI-S), which measures situational and transient anxiety, and the Trait Anxiety Scale (STAI-T), which captures a stable predisposition to experience anxiety across situations and over time. In the present study, only the STAI-T was administered, focusing on participants’ general anxiety proneness. Items are rated on a four-point Likert scale, with higher scores reflecting greater trait anxiety. The STAI-T has demonstrated excellent internal consistency and good construct validity and is widely used in clinical and research settings [41].

Although no universally accepted diagnostic cutoffs are established for the STAI-T, previous studies suggest that scores of 45 or higher may reflect high levels of anxiety, scores between 38 and 44 moderate anxiety, and scores of 37 or below low anxiety. These thresholds should nevertheless be interpreted with caution, taking into account population-specific norms and clinical context [41].

Emotional dysregulation was assessed using the Difficulties in Emotion Regulation Scale (DERS). The DERS was used to assess habitual, trait-like difficulties in emotion regulation rather than momentary or state-dependent emotional responses. This self-report questionnaire consists of 36 items evaluating six specific dimensions: non-acceptance of negative emotions, difficulties engaging in goal-directed behavior when distressed, impulse control difficulties, lack of emotional awareness, limited access to effective regulation strategies, and lack of emotional clarity. Each item is rated on a 5-point Likert scale ranging from 1 (almost never) to 5 (almost always), with higher scores indicating greater emotional dysregulation.

The six DERS subscales are as follows:

- Nonacceptance of negative emotions (DERS Non-Acceptance):

Evaluates the tendency to reject, judge, or feel ashamed of one’s negative emotions. High scores indicate difficulty accepting emotional experiences without judgment, thereby exacerbating distress.

- Difficulties engaging in goal-directed behavior (DERS Goals):

Measures the ability to stay focused and productive when distressed. High scores suggest that negative emotions interfere with daily functioning, especially in stressful contexts.

- Impulse control difficulties (DERS Impulse):

Assesses the inability to inhibit impulsive behaviors in response to negative emotions. High scores indicate a loss of behavioral control under emotional distress.

- Lack of emotional awareness (DERS Awareness):

Evaluates attentiveness to one’s current emotional state. High scores reflect emotional disengagement or difficulty recognizing the presence of emotions.

- Limited access to emotion regulation strategies (DERS Strategies):

Reflects the belief that nothing can be done to alleviate distress. High scores indicate a sense of emotional helplessness.

- Lack of emotional clarity (DERS Clarity):

Assesses the ability to clearly identify and differentiate one’s emotions. High scores indicate emotional confusion [23].

The French version of the DERS has been validated by two independent research teams. Côté et al. (2013) confirmed the six-factor structure in a French-speaking adult sample and reported high overall internal consistency (α = 0.94), with Cronbach’s alpha coefficients ranging from 0.74 to 0.90 across subscales and satisfactory temporal stability [42].

Similarly, Dan-Glauser and Scherer (2013) [43] validated the DERS in a French-speaking university population, observing excellent internal consistency (α = 0.92) and high test–retest reliability (r = 0.88, *p* < 0.001) over a four-week period. Factor congruence between the original and French versions was estimated at 0.98 (Tucker’s phi), confirming strong structural equivalence. Subscale alphas were 0.87 for Nonacceptance, 0.90 for Goals, 0.87 for Impulse, 0.80 for Awareness, 0.87 for Strategies, and 0.74 for Clarity [43].

These findings support the validity and reliability of the French version of the DERS for clinical and research use, particularly among adolescent and young adult populations.

Impulsivity was measured using the French short form of the UPPS-P Impulsive Behavior Scale. This self-administered instrument consists of 20 items assessing five theoretically distinct facets of impulsivity: Negative Urgency, defined as the tendency to act impulsively in response to negative emotional states; Positive Urgency, reflecting impulsive reactions during intense positive affect; Lack of Premeditation, referring to reduced consideration of consequences prior to action; Lack of Perseverance, indicating difficulties sustaining attention or effort on tasks; and Sensation Seeking, which captures the inclination toward novel, exciting, or stimulating experiences.

Responses are provided on a four-point Likert scale ranging from 1 (strongly agree) to 4 (strongly disagree), with higher scores indicating greater impulsivity. Psychometric evaluations of the French version have shown satisfactory internal consistency across francophone samples, with Cronbach’s alpha coefficients of 0.78 for Negative Urgency, 0.70 for Positive Urgency, 0.79 for Lack of Premeditation, 0.84 for Lack of Perseverance, and 0.83 for Sensation Seeking. Reliability indices for the full UPPS-P are comparable (approximately 0.77–0.83), and the short version has demonstrated excellent test–retest reliability, with correlation coefficients ranging from 0.84 to 0.92 [44].

In addition to psychometric assessments, participants completed a standardized self-report questionnaire collecting sociodemographic and clinical information. Recorded variables included sex, age, educational attainment, student status, medical or surgical history, presence of psychiatric comorbidities, current psychotropic treatment, and ongoing psychological care. Further data were obtained on health-related and risk behaviors, including tobacco use, alcohol consumption, and illicit substance use, as well as the presence, frequency, and history of suicide attempts and family history of psychiatric disorders.

### 2.3. Statistical Analyses

All analyses were performed using Stata statistical software (version 14; StataCorp LLC, College Station, TX, USA). The sample was divided into two groups: a non-clinical group and a clinical group composed of participants diagnosed with BPD.

Descriptive statistics for categorical variables are reported as absolute frequencies and percentages, with between-group comparisons carried out using the chi-square test. Continuous variables are presented as medians with interquartile ranges (25th–75th percentiles), and group differences were examined using the Wilcoxon–Mann–Whitney nonparametric test.

To investigate group-related differences in emotion regulation, quantile regression models were applied to DERS subscale scores. These analyses controlled for potential confounders that differed significantly between groups, including sex, personal psychiatric history, presence of psychiatric comorbidities, substance use, family history of psychiatric disorders, number of suicide attempts, previous and current psychological follow-up, psychotropic medication use, and levels of depressive and anxiety symptoms as measured by the BDI-II and STAI-T.

Subsequently, multivariate logistic regression analyses were conducted to assess whether DERS dimensions that remained significant in the adjusted quantile regressions were independently associated with BPD status. The Impulse, Awareness, and Clarity subscales, as well as the total DERS score, were entered into the models. All logistic regression analyses were adjusted for sex, depressive symptom severity (BDI-II score), and trait anxiety (STAI-T score).

In line with established recommendations for multivariate logistic regression, ensuring a minimum of 10 observations per covariate, both groups included a sufficient number of participants (≥40) to support the validity of the analyses.

Model calibration and specification were evaluated using the Hosmer–Lemeshow goodness-of-fit test and the Link test.

Finally, within the BPD group, Spearman’s rank-order correlation coefficients were computed to examine associations between DERS subscales, dimensions of borderline symptomatology assessed by the DIB-R, relevant clinical variables (including suicide attempts and substance use), and impulsivity traits measured with the UPPS-P.

### 2.4. Ethics Statement

This research protocol was approved by the Ethics Committee of the Faculty of Medicine, Erasme Hospital—Université Libre de Bruxelles (ULB), Brussels, Belgium (reference: P2020/111). Written informed consent was obtained from all participants aged 18 years or older, and from their legal guardians or parents for participants under the age of 18.

## 3. Results

### 3.1. Sociodemographic and Clinical Characteristics of the Sample

The sample consisted of 184 participants, divided into a non-clinical group (*n* = 140) and a group of individuals diagnosed with BPD (*n* = 44). A significantly higher proportion of women was observed in the BPD group compared with the non-clinical group (95.4% vs. 73.6%; *p* = 0.002).

Personal psychiatric history was significantly more frequent among participants with BPD (45.4%) than among non-clinical group (2.9%; *p* < 0.001), as were psychiatric comorbidities: none of the non-clinical participants presented comorbid disorders, whereas 68.2% of BPD participants had one or more comorbidities (*p* < 0.001). Likewise, illicit substance use was markedly higher in the BPD group (47.7%) compared with the non-clinical group (4.3%; *p* < 0.001).

Family history of psychiatric disorders was reported by 88.6% of BPD participants versus 14.3% of non-clinical group (*p* < 0.001). Regarding suicidal behaviors, 77.3% of individuals in the BPD group reported having attempted suicide at least once, compared with only 6.4% in the non-clinical group (*p* < 0.001). Past and current psychological follow-up were also significantly more frequent among BPD participants (79.5% and 100%, respectively) than among non-clinical group (9.3% and 0%; *p* < 0.001 for both comparisons). Similarly, psychotropic medication use was reported by 72.7% of BPD participants and by none of the non-clinical group (*p* < 0.001).

In contrast, no significant differences were found between groups regarding student status (*p* = 0.643), medical or surgical history (*p* = 0.693), alcohol consumption (*p* = 0.145), or educational level (*p* = 0.158).

Among continuous variables, median age did not differ significantly between groups (*p* = 0.056). However, BPD participants reported a significantly higher number of suicide attempts (*p* < 0.001) (Table 1).

On a psychometric level, participants with BPD displayed significantly higher scores on all DERS subscales—Nonacceptance of negative emotions, Difficulties engaging in goal-directed behavior when distressed, Impulse control difficulties, Lack of emotional awareness, Limited access to effective regulation strategies, and Lack of emotional clarity—as well as on the total DERS score (*p* < 0.001 for all comparisons). BDI and STAI-T scores were also significantly higher in the BPD group (*p* < 0.001 for both) (Table 2).

Regarding impulsivity dimensions assessed by the UPPS-P, individuals with BPD scored significantly higher on Negative Urgency, Positive Urgency, Lack of Premeditation, and Lack of Perseverance (*p* < 0.001 for each), whereas no significant difference was found for Sensation Seeking (*p* = 0.119) (Table 2).

The intensity of borderline pathology within the clinical sample was evaluated using the Diagnostic Interview for Borderlines—Revised (DIB-R). Median values for each of the four domains—affective, cognitive, impulsive, and interpersonal functioning—as well as the overall DIB-R score, are reported in Table 3.

### 3.2. Quantile Regression Analysis

Group differences in emotion regulation difficulties were examined using quantile regression models adjusted for the DERS subscale scores. These models assessed the extent to which belonging to the BPD group was associated with increased emotional regulation difficulties, while controlling for the following potential confounders: gender, psychiatric history, psychiatric comorbidities, substance use, family history of psychiatric disorders, number of suicide attempts, past and current psychological follow-up, psychotropic medication, BDI-II score, and STAI-T score.

Results are presented in Table 4. Several DERS dimensions remained significantly associated with BPD status after adjustment. In particular, the Impulse dimension showed an adjusted coefficient of 8.1 (standard error = 3.2), indicating a marked increase in impulsivity-related difficulties among individuals with BPD, independent of confounding variables. Similarly, lack of emotional Awareness was significantly higher, with an adjusted coefficient of 10.5 (SE = 2.8), as was lack of emotional Clarity (adjusted coefficient = 6.2, SE = 2.2).

Moreover, the total DERS score was significantly higher in participants with BPD, with an adjusted coefficient of 37.4 (SE = 11.4; *p* < 0.05), reflecting a more pervasive emotional dysregulation beyond specific dimensions.

In contrast, Nonacceptance, Goals, and Strategies dimensions did not reach statistical significance in the adjusted models, although coefficients indicated a trend toward higher scores in the BPD group.

Overall, these analyses confirm that specific facets of emotional dysregulation—particularly emotional impulsivity and deficits in emotional awareness and clarity—represent robust differentiating markers of BPD in young adults, independent of psychopathological and contextual factors.

### 3.3. Multivariate Logistic Regression Analysis

A multivariate logistic regression analysis was conducted to determine which dimensions of emotion regulation were most strongly associated with belonging to the BPD group, while controlling for the following clinical and demographic covariates: gender, depressive symptoms (BDI-II score), and trait anxiety (STAI-T score).

As shown in Table 5, several DERS dimensions emerged as independent predictors of BPD diagnosis. In particular, participants scoring above 15 on the Impulse subscale were 5.91 times more likely to belong to the BPD group (adjusted OR = 5.91; 95% CI: 2.27–15.37; *p* < 0.001). Similarly, a score above 17 on the lack of emotional Awareness subscale was associated with a 3.56-fold increase in the likelihood of BPD classification (adjusted OR = 3.56; 95% CI: 1.52–8.35; *p* = 0.003), while a score above 15 on lack of emotional Clarity also significantly predicted group membership (adjusted OR = 2.90; 95% CI: 1.25–6.73; *p* = 0.013).

Finally, participants with a total DERS score above 129 were 12.08 times more likely to belong to the BPD group (adjusted OR = 12.08; 95% CI: 4.54–32.16; *p* = 0.008), confirming the strong discriminative value of global emotional dysregulation in identifying individuals with BPD.

Taken together, these analyses indicate that specific components of emotion regulation—particularly emotional impulsivity, emotional awareness, and emotional clarity—represent powerful and independent markers of BPD in young adults, further underscoring the central role of emotional dysregulation in the clinical characterization of BPD.

### 3.4. Correlations Between Emotional Dysregulation and Clinical Dimensions of Borderline Personality Disorder

Spearman’s correlation analyses were conducted to examine the relationships between dimensions of emotional dysregulation (DERS) and the clinical components of the Diagnostic Interview for Borderlines—Revised (DIB-R), as well as depressive symptoms (BDI-II) and trait anxiety (STAI-T), among participants diagnosed with BPD (n = 44).

As shown in Table 6, several significant associations were observed between DERS dimensions and clinical variables in the BPD group. The Impulse dimension of the DERS was positively correlated with the total DIB-R score (r = 0.317; *p* < 0.05), suggesting that greater difficulty in controlling emotional impulses is associated with higher overall borderline symptom severity. Moreover, Nonacceptance of emotions was significantly correlated with the affective dimension of the DIB-R (r = 0.312; *p* < 0.05), underscoring the role of negative emotion rejection in the emotional instability characteristic of BPD.

The Strategies dimension showed significant positive correlations with the total DIB-R score (r = 0.344; *p* < 0.05), the interpersonal dimension (r = 0.342; *p* < 0.05), as well as with depressive symptoms (BDI-II, r = 0.502; *p* < 0.05) and trait anxiety (STAI-T, r = 0.521; *p* < 0.05). These findings indicate that individuals who experience greater difficulty accessing effective emotion regulation strategies also exhibit more pronounced interpersonal dysfunction, alongside heightened emotional distress across borderline, depressive, and anxious domains.

Additionally, the Goals dimension of the DERS was significantly correlated with both depressive (r = 0.350; *p* < 0.05) and trait anxiety scores (r = 0.301; *p* < 0.05), suggesting that difficulties maintaining goal-directed behavior under emotional stress are closely linked to mood and anxiety disturbances.

Notably, the Awareness dimension was negatively correlated with the cognitive dimension of the DIB-R (r = −0.404; *p* < 0.05), indicating that individuals with lower emotional awareness are less likely to exhibit the cognitive distortions typically associated with BPD.

Finally, the total DERS score was positively correlated not only with depression (r = 0.354; *p* < 0.05) and trait anxiety (r = 0.438; *p* < 0.05), but also with the total DIB-R score (r = 0.309; *p* < 0.05), highlighting the cross-cutting role of global emotional dysregulation in the symptomatic expression of mood, anxiety, and borderline psychopathology.

### 3.5. Correlations Between Emotional Dysregulation, Suicidal Behavior, and Substance Use

Spearman’s correlation analyses were conducted to explore the relationships between dimensions of DERS and several clinical markers—namely, the number of suicide attempts, history of suicide attempts, and substance use—among participants in the BPD group.

As shown in Table 7, most correlations between DERS subscales and these clinical variables were not statistically significant, suggesting a relative independence between self-reported emotional difficulties and certain objectively observable impulsive behaviors. However, a significant negative correlation was observed between lack of emotional Clarity and the number of suicide attempts (r = −0.386; *p* < 0.05). This association should be interpreted with caution, as the number of suicide attempts represents a cumulative lifetime measure, whereas emotional clarity reflects current subjective functioning.

No other DERS dimensions were significantly correlated with suicide attempt history or substance use. These findings suggest that, within this sample, the associations between emotional dysregulation and behavioral impulsivity are limited, and may be specific to certain emotional facets—particularly emotional clarity deficits in relation to suicidal behaviors.

### 3.6. Correlations Between Emotion Regulation and Impulsivity Traits Measured by the UPPS-P

Spearman’s correlation analyses were conducted to examine the relationships between DERS and UPPS-P among participants with BPD.

As shown in Table 8, several DERS dimensions were significantly associated with specific facets of impulsivity. In particular, the Impulse dimension of the DERS was positively correlated with both Negative Urgency (r = 0.544; *p* < 0.05) and Positive Urgency (r = 0.345; *p* < 0.05), suggesting that difficulties in regulating emotional impulses are closely linked to heightened reactivity to intense emotions, whether negative or positive.

Furthermore, the total DERS score was significantly correlated with several impulsivity traits, including Negative Urgency (r = 0.422; *p* < 0.05), Positive Urgency (r = 0.380; *p* < 0.05), and Lack of Premeditation (r = 0.423; *p* < 0.05), reflecting an interdependence between global emotional dysregulation and the tendency to act without forethought in emotionally charged contexts.

The Strategies dimension of the DERS also showed a significant correlation with Positive Urgency (r = 0.334; *p* < 0.05), indicating that individuals with limited access to adaptive regulation strategies are more likely to exhibit increased impulsivity in response to positive affective states.

Additional significant associations were found between lack of emotional Awareness and both Lack of Premeditation (r = 0.376; *p* < 0.05) and Lack of Perseverance (r = 0.423; *p* < 0.05), suggesting that reduced awareness of emotional states is associated with difficulties in planning and maintaining goal-directed behavior.

In contrast, no significant correlations were observed with the Sensation Seeking dimension.

Overall, these findings suggest that in individuals with BPD, specific facets of emotional dysregulation—particularly emotional impulsivity, limited access to adaptive strategies, low emotional awareness, and global dysregulation—are closely related to characteristic impulsivity profiles of the disorder, notably those centered on emotional urgency and executive control failures such as poor premeditation and perseverance.

## 4. Discussion

### 4.1. General Context and Specificity of Borderline Personality Disorder During the Transitional Age

The present study falls within the field of transition psychiatry, a developmental period extending from late adolescence to early adulthood, during which emotional regulation and impulse control continue to mature. This phase is recognized as particularly vulnerable to the expression of borderline symptomatology [9].

From this developmental perspective, the main objective of this study was to better characterize the profile of emotional dysregulation among young adults with BPD, using DERS as both a theoretical and assessment framework. Our central hypothesis was that individuals with BPD would exhibit significantly greater difficulties across all DERS dimensions compared to a non-clinical group. We further hypothesized that specific dimensions of emotion regulation would represent robust predictors of BPD diagnosis, independent of comorbid anxiety and depressive symptomatology.

In addition, we explored the associations between these emotion regulation dimensions and key clinical variables, including DIB-R dimensions, suicidal behaviors, substance use, and impulsivity traits.

These findings should, however, be interpreted in light of the developmental context characteristic of this age range, in which the maturation of emotional and cognitive systems remains incomplete. This framework provides a meaningful lens for understanding the observed regulation difficulties, consistent with Chapman’s (2019) model [16]. According to this model, the asynchronous maturation between heightened limbic reactivity and still-developing prefrontal control increases young individuals’ vulnerability to emotional distress and promotes reliance on immediate regulation strategies, which are often impulsive or self-damaging [16].

Thus, emotional dysregulation emerges as a dynamic phenomenon, closely tied to the stage of development and modulated by environmental factors. It should be considered in continuous interaction with its contextual expression. Within this framework, identifying specific markers associated with BPD carries substantial clinical and preventive relevance. Nonetheless, the transition to adulthood represents a highly heterogeneous period, characterized by wide interindividual variability in the pace and intensity of neurodevelopmental and psychosocial transitions, which may contribute to the diversity of profiles observed in this age group [9].

#### 4.1.1. Sociodemographic Characteristics

The results revealed several significant differences between participants with BPD and non-clinical subjects on sociodemographic and clinical variables, partially confirming patterns previously identified in similar samples.

In our cohort, the BPD group did not differ significantly from the non-clinical group in terms of age or educational level, with the majority of participants being students. This confirms a basic level of homogeneity between groups and indicates that the demographic characteristics of our sample are broadly consistent with those reported in the literature on BPD.

As is frequently observed in clinical studies, the majority of participants in the BPD group were female (95.4%), representing a significantly higher proportion than in the non-clinical group (83.5%). This strong female predominance is consistent with classical epidemiological data reporting a female-to-male ratio of approximately 3:1 [18,19].

However, this overrepresentation may not necessarily reflect a true sex-based difference in prevalence, but could instead result from contextual, methodological, and sociocultural factors. Several hypotheses have been proposed to explain this recurrent bias: a greater tendency among women to seek psychological help and to participate in clinical studies; heightened clinical sensitivity to internalizing symptoms typically expressed by women; and conversely, an under-recognition of externalizing manifestations of BPD in men, who are more frequently classified under alternative diagnoses such as conduct disorders or substance use disorders [45,46].

In line with this interpretation, population-based studies and recent meta-analytic evidence suggest a more balanced prevalence of BPD across sexes in the general population [45]. Thus, the female predominance observed in our cohort aligns with a well-documented trend in the literature, while also underscoring the need for a nuanced interpretation that integrates factors such as socialization processes, sampling biases, and sex-specific patterns of symptom expression [18].

#### 4.1.2. Psychiatric Comorbidities and Clinical History

Our study confirms that young adults with BPD are characterized by a significant psychopathological burden, reflected in the high frequency of psychiatric comorbidities, greater use of psychological care, and higher rates of substance use compared to the non-clinical group.

Specifically, 68.2% of participants with BPD presented at least one psychiatric comorbidity (versus 0% of non-clinical group), and 45.4% reported a personal psychiatric history (versus 2.9%). In addition, the prevalence of familial psychiatric history reached 88.6% in the BPD group, compared with 14.3% among non-clinical group, suggesting a multifactorial vulnerability encompassing biological, familial, and environmental components.

These findings are consistent with previous research [46,47], which emphasize the central role of developmental and familial factors in the etiology of BPD—particularly early exposure to parental psychopathology, addictive behaviors, or dysfunctional family climates. Familial history often includes paternal substance use or maternal borderline traits, supporting the hypothesis of an intergenerational transmission of psychopathological vulnerability, encompassing emotional regulation deficits, attachment difficulties, and risk-taking behaviors [46,48].

The high proportion of participants currently receiving psychological care (100%) or with past psychotherapeutic follow-up (79.5%), together with the elevated prevalence of psychotropic medication use (72.7%), further illustrates the substantial psychological distress experienced by these young adults.

No significant differences were found regarding somatic comorbidities. This result can likely be interpreted in light of the early developmental stage of the sample (median age: 18 years), a period during which the physical consequences of risky behaviors or BPD-related eating disturbances often remain limited. Such somatic complications tend to emerge later in adulthood, when problematic behaviors become more chronic and cumulative [49].

#### 4.1.3. Risk Behaviors and Suicidality

Substance use (cannabis, and other illicit drugs) was significantly higher in the BPD group, with 47.7% of participants reporting substance use compared to 4.3% in the non-clinical group. This finding is consistent with existing literature describing addictive behaviors as a common form of emotional self-regulation among individuals with BPD [18]. Several authors, including Mungo et al. (2025) and Bohus et al. (2021), emphasize that these behaviors do not solely reflect behavioral impulsivity, but often represent maladaptive strategies for coping with emotional distress [37,46].

The prevalence of suicidal behaviors in our sample was particularly high, underscoring the clinical severity of BPD. In the BPD group, 77.3% of participants reported at least one suicide attempt, compared with only 6.4% in the non-clinical group—a highly significant difference. This striking proportion is consistent with recent population-based and meta-analytic evidence indicating that BPD is strongly associated with suicide attempts and represents one of the psychiatric conditions most robustly linked to suicidal behavior in the general population [45].

Beyond prevalence alone, participants with BPD also reported a significantly higher number of suicide attempts, highlighting the recurrent and chronic nature of these self-injurious behaviors. These results are consistent with recent findings by Fortaner-Uyà et al. (2025), demonstrating that the repetition of suicide attempts constitutes a prognostic indicator of clinical severity and poor outcome, particularly among young adults with BPD [7].

From this perspective, suicidal behaviors in young adults with BPD should not be viewed as isolated events but rather as the expression of an extreme and dysfunctional emotion regulation pattern. Their frequency and recurrence underscore the importance of early therapeutic interventions targeting distress tolerance, emotional regulation, and crisis prevention, as promoted by integrative approaches such as Dialectical Behavior Therapy (DBT) [18,46].

#### 4.1.4. Anxious–Depressive Comorbidity and Impulsivity

Psychometric analyses confirmed the clinical and subjective severity of the profile observed among young adults with BPD. Participants in the BPD group scored significantly higher on both the BDI-II and the STAI-T compared to the non-clinical group, indicating marked anxious–depressive comorbidity and chronic emotional distress. These findings are consistent with a large body of literature describing BPD as a disorder of high emotional comorbidity, frequently associated with severe depressive and anxious symptoms [18,50,51].

The mean BDI-II scores among BPD participants reached a “severe” level, reflecting profound psychological suffering and intense depressive affect. This finding has major clinical implications, as depressive symptoms are highly prevalent in BPD and contribute to an increased risk of suicidal behavior, within a broader clinical profile characterized by elevated suicidality [7,45,52]. However, mood symptoms in this context differ from those seen in unipolar depression, as they are typically characterized by interpersonal reactivity—with emotional fluctuations frequently triggered by relational stressors, complicating both assessment and treatment [27,53].

Similarly, elevated STAI-Trait scores indicate a stable vulnerability to anxiety, independent of situational stress. This dispositional tendency may play a critical role in amplifying emotional reactivity and behavioral impulsivity. These observations align with contemporary models of BPD, which conceptualize the disorder as involving persistent emotional hyperreactivity and a low threshold for negative affect tolerance, often associated with significant anxious comorbidity [18,54].

These findings are also in line with evidence showing that young adults with BPD exhibit increased comorbidity with mood disorders, anxiety disorders, and ADHD, contributing to the complexity of clinical management and the variability of illness trajectories [18,45,46]. They further highlight the need for multimodal, individualized therapeutic strategies addressing emotional dysregulation, risk behaviors, and anxious–depressive comorbidities simultaneously.

In terms of impulsivity, the results confirmed the presence of a marked and multidimensional impulsivity profile among young adults with BPD. On the UPPS-P, individuals with BPD scored significantly higher on Negative Urgency, Positive Urgency, Lack of Premeditation, and Lack of Perseverance (*p* < 0.001 for each), while no significant difference was observed for Sensation Seeking (*p* = 0.119). This pattern indicates that the impulsive profile in BPD is dominated by emotional urgency, reflecting a tendency to act in a disinhibited manner under intense emotional states, whether negative or positive. This profile of emotional impulsivity is consistent with the findings of Mungo et al. (2025) [37].

Among these dimensions, Negative Urgency emerged as the most pronounced, confirming its central role in BPD functioning. This form of affective impulsivity represents a key behavioral manifestation of emotional dysregulation and is frequently associated with self-destructive behaviors such as self-injury, substance use, and suicide attempts [37].

The concomitant elevation of Positive Urgency further suggests that impulsive reactivity in BPD is not limited to aversive emotions, but can also be triggered by intense positive affects such as excitement or euphoria—reflecting a global imbalance in emotion regulation processes [37].

Moreover, elevated scores on Lack of Premeditation and Lack of Perseverance suggest difficulties in anticipating the consequences of actions and in maintaining goal-directed behavior in emotionally charged contexts. These dimensions, which are more closely related to executive control than to affective reactivity, may contribute to the behavioral disorganization observed in young adults with BPD. However, recent evidence indicates that these cognitive control facets lose their diagnostic specificity once anxiety and depressive symptoms are controlled for, suggesting that they may reflect secondary effects of emotional overload rather than core features of the disorder [37].

Convergently, Mungo et al. (2025) demonstrated that the affective dimensions of impulsivity assessed by the UPPS-P—particularly Negative Urgency and Positive Urgency—were the most discriminant markers of BPD in young adults, while Sensation Seeking contributed little to diagnostic differentiation [37]. Likewise, Sebastian et al. (2013) emphasized that impulsivity in BPD cannot be reduced to a simple executive control deficit, but rather reflects a broader emotional dysfunction, in which urgency dimensions play a central role [25]. These authors argue that traditional behavioral measures often fail to capture this affective component of impulsivity, advocating instead for the use of integrative models, such as the UPPS-P, to better identify clinically specific impulsivity profiles [25].

Taken together, these findings confirm the existence of a multidimensional emotional and cognitive vulnerability: young adults with BPD exhibit high depressive intensity, heightened anxiety reactivity, and marked affective impulsivity. This profile supports the view that BPD is not merely characterized by emotional instability but rather reflects a broader imbalance of affective and cognitive regulatory systems, in which the interaction between impulsivity, anxiety, and depressive distress plays a determining role in the clinical expression of the disorder [37].

### 4.2. Psychometric Profile: Emotional Dysregulation as the Core Feature of BPD

#### 4.2.1. Emotional Dysregulation as a Core Mechanism: Empirical and Conceptual Perspectives

The results obtained on the Difficulties in Emotion Regulation Scale (DERS) reveal marked impairments across all dimensions of emotion regulation among young adults with BPD. Participants in the BPD group scored significantly higher on every DERS subscale and on the total score (*p* < 0.001), indicating a generalized emotional dysregulation. This dysregulation encompasses emotion recognition and acceptance, emotional clarity, difficulties maintaining goal-directed behavior under emotional distress, impulse control problems in affectively charged contexts, and limited access to adaptive regulation strategies.

These findings support the hypothesis that young adults with BPD do not merely struggle to accept or modulate their emotions, but also exhibit profound impairments in perceiving, identifying, and understanding internal emotional states. This global deficit—originally described by Gratz and Roemer (2004) [23] and later confirmed in adolescents <by Ibraheim et al. (2017) [4]—fits within a multisystemic framework of emotional dysregulation, affecting perceptual, evaluative, and behavioral processes involved in emotional responding [27].

Conceptually, these results are consistent with Linehan’s biosocial model, which posits that a constitutional emotional vulnerability, when combined with an invalidating environment, fosters the development of a deficient emotion regulation style typical of BPD [11]. This framework has been strengthened by several empirical studies, highlighting that difficulties in identifying, accepting, and managing emotions constitute a core psychopathological feature of BPD from early adulthood [4,27].

The global elevation of DERS scores in our sample thus indicates that emotion regulation deficits are not secondary symptoms, but rather structural characteristics of borderline functioning [4,27]. In line with the meta-analysis by Daros & Williams (2019), our data suggest that the combination of limited use of adaptive strategies, difficulty maintaining goal-directed behavior under emotional stress, and heightened affective impulsivity represents a particularly deleterious configuration for psychological stability and the prevention of risk behaviors [27].

#### 4.2.2. Quantile Regression Analysis: Key Predictive Dimensions of Borderline Personality Functioning

Quantile regression analyses were conducted to further delineate the specific nature of the difficulties observed. After adjustment for potential confounding variables, several DERS subdimensions remained positively and significantly associated with membership in the BPD group: emotional impulsivity (β = 8.1; SE = 3.2; *p* < 0.05), lack of emotional awareness (β = 10.5; SE = 2.8; *p* < 0.05), and lack of emotional clarity (β = 6.1; SE = 2.2; *p* < 0.05). The total DERS score also remained significantly associated with BPD diagnosis (β = 37.4; SE = 11.4; *p* < 0.05), indicating a globally higher level of emotional dysregulation independent of comorbid affective symptoms.

In contrast, the Nonacceptance, Goals, and Strategies dimensions did not retain statistical significance in the adjusted models, although a trend toward higher scores in the BPD group persisted. Overall, these findings suggest that deficits in emotional identification and clarity, together with impaired impulse inhibition in affective contexts, represent the most discriminant components of borderline functioning at this developmental stage.

These results resonate with the longitudinal findings of Lee, Keng, and Hong (2024), which provide empirical support for the biosocial model [15]. The authors demonstrated that impulsivity and emotional vulnerability significantly predicted emotional dysregulation and BPD symptom severity over a six-month period, independently of the degree of parental invalidation. Moreover, their study identified a bidirectional relationship between emotional vulnerability and invalidation, suggesting the existence of a transactional vicious cycle: the adolescent’s distress elicits invalidating parental responses, which in turn exacerbate emotion regulation difficulties [15]. This process helps to elucidate how deficits in emotional recognition and understanding interact with impulsive stress responses, thereby reinforcing the biosocial architecture underlying the development of BPD during adolescence.

#### 4.2.3. Multivariate Logistic Regression: The Predictive Triad

The multivariate logistic regression analysis identified the DERS subdimensions most strongly associated with a diagnosis of BPD after adjusting for sex, depressive symptoms (BDI-II), and trait anxiety (STAI-T).

Difficulties in impulse control (adjusted OR = 5.91; 95% CI [2.27–15.37]; *p* < 0.001), lack of emotional awareness (adjusted OR = 3.56; 95% CI [1.52–8.35]; *p* = 0.003), and lack of emotional clarity (adjusted OR = 2.90; 95% CI [1.25–6.73]; *p* = 0.013) emerged as the most robust predictors of BPD diagnosis.

In addition, a total DERS score above 129 was associated with a twelvefold increase in the likelihood of belonging to the BPD group (adjusted OR = 12.08; 95% CI [4.54–32.16]; *p* = 0.008), confirming the strong discriminative value of global emotional dysregulation.

These findings support the hypothesis that emotional dysregulation is not merely a secondary consequence of anxious or depressive symptomatology, but rather a specific and central process underlying BPD.

#### 4.2.4. From Emotional Impulsivity to Cognitive Dysregulation: The Impulse–Awareness–Clarity Triad as a Core Mechanism in BPD

The pattern of emotional dysregulation observed in our study aligns closely with the multidimensional model proposed by Gratz and Roemer (2004) [23], which conceptualizes emotion regulation as involving interdependent processes of identification, understanding, and modulation of emotional states. Difficulties in recognizing, differentiating, and accepting one’s emotions impair behavioral control, thereby fostering impulsive responses under stress [23].

Consistent with this view, several empirical studies have emphasized the central role of specific DERS dimensions—particularly Impulse, Awareness, and Clarity—in distinguishing individuals with borderline traits or diagnoses from healthy controls. In a non-clinical sample of Italian adolescents, Fossati et al. (2013) found that the subscales Impulse, Strategies, and Clarity significantly differentiated participants with high borderline traits from controls, suggesting that emotional dysregulation represents an early marker of borderline functioning [29]. However, unlike our clinical sample of treated or hospitalized young adults, their study was conducted in a community population, which may explain some contextual discrepancies.

In our study, although the Strategies subscale did not reach statistical significance after adjustment, its mean elevation within the BPD group likely reflects a tendency to rely on regulatory strategies perceived as ineffective. This attenuation may be related to the therapeutic engagement of most participants, who had already received interventions aimed at improving emotion regulation skills, thereby reducing intergroup variability.

Comparable results were reported by Salgó et al. (2021) in a clinical sample of adults with BPD, who exhibited significantly higher scores on all DERS subscales relative to controls, with the largest differences found for Goals, Impulse, and Strategies [55]. This pattern supports the notion of generalized emotional dysregulation, dominated by difficulties in maintaining goal-directed behavior and implementing adaptive strategies during affective distress [55].

Similarly, Rufino et al. (2017) identified among hospitalized adults a Global Dysregulation profile characterized by elevated Impulse and Clarity scores, which were associated with greater suicidality, higher functional impairment, and poorer symptomatic improvement during treatment [56]. These findings converge with ours, highlighting a specific Impulse–Awareness–Clarity triad that reflects both behavioral disinhibition and cognitive disorganization in emotional processing.

Further supporting this framework, Ibraheim et al. (2017) demonstrated that adolescents with BPD scored significantly higher on the total DERS, particularly on Impulse and Strategies, even after controlling for age, sex, and psychiatric severity [4]. These data underscore the robustness of these dimensions across developmental stages and independent of affective comorbidities. In line with this, Waite et al. (2024) found that negative and positive emotional dysregulation contribute differentially to impulsive behaviors—negative dysregulation fostering disinhibition under distress, and positive dysregulation promoting sensation seeking—thereby illustrating the transdiagnostic role of emotion dysregulation in risk-taking behaviors [30].

Within this broader context, our findings reinforce the importance of emotional awareness and clarity, two often underemphasized yet conceptually pivotal components. Elevated Awareness scores indicate a confused or disorganized perception of internal states, while deficits in Clarity reflect difficulty interpreting and labeling emotional experiences. Such impairments hinder access to reflective processes necessary for emotional modulation and are associated with chronic affective instability [8]. As Gratz and Roemer (2004) and Ibraheim et al. (2017) highlight, lack of awareness of internal states amplifies affective and interpersonal dysfunction, perpetuating maladaptive regulatory cycles [4,23].

This interpretation is further supported by meta-analytic evidence showing that reduced emotional awareness and alexithymic traits are moderately associated with BPD, while also underscoring the methodological challenges of assessing emotional processes predominantly through self-report measures [57].

More broadly, the combination of low clarity and awareness, heightened emotional impulsivity, and limited perceived efficacy of regulation strategies delineates a developmental vulnerability profile characteristic of emerging BPD. Emotionally reactive yet strategically under-equipped, these individuals appear particularly at risk for chronic dysregulation, justifying early clinical interventions targeting both the emotional and cognitive components of regulation.

From a developmental standpoint, our results converge with Wyrzykowski et al. (2025), who showed that lack of emotional clarity predicts risk-taking behaviors among individuals with BPD, supporting its role as a transdiagnostic marker of behavioral dyscontrol [58]. Similarly, Porter et al. (2016) found that differences between individuals with high versus low borderline traits did not concern the types of regulation strategies used, but rather the efficiency and contextual appropriateness of their use, particularly under high emotional intensity [59]. This supports the view that the core deficit in BPD lies not in the strategic repertoire itself, but in the metacognitive capacities of awareness and clarity required for adaptive emotion regulation [59].

Taken together, these converging findings suggest that emotional impulsivity, reduced awareness, and low clarity form an interdependent triad that underpins the core mechanism of borderline emotional dysregulation. The transition from behavioral impulsivity to cognitive dysregulation encapsulates the developmental shift observed in emerging adulthood, where deficits in identifying and integrating affective experiences become increasingly crystallized, sustaining the chronic emotional and interpersonal instability that defines BPD.

### 4.3. Correlations Between Emotional Dysregulation and Clinical Dimensions of Borderline Personality Disorder

#### 4.3.1. Significant Correlations Between DERS and Clinical Dimensions

Spearman correlation analyses conducted within the BPD group revealed several significant associations between the subdimensions of the DERS and the clinical components of the DIB-R.

The Impulse subscale of the DERS was positively correlated with the total DIB-R score (r = 0.317; *p* < 0.05), indicating that greater difficulties in controlling emotional impulses are associated with higher overall BPD severity. This finding is consistent with the longitudinal results of Lee et al. (2024), who showed that emotional impulsivity significantly predicts the intensity of borderline symptoms at six months, suggesting that it constitutes a dynamic and cross-cutting component of the disorder [15].

The Nonacceptance subscale was positively correlated with the affective dimension of the DIB-R (r = 0.312; *p* < 0.05), suggesting that difficulties tolerating or validating one’s own negative emotions—such as shame, guilt, or sadness—may contribute to the marked affective instability characteristic of BPD. This finding echoes Azad et al. (2025), who demonstrated that mentalization deficits, particularly uncertainty regarding one’s own and others’ mental states, are associated with greater emotional distress and dysfunctional regulation behaviors (e.g., substance use) among youths with borderline traits [60].

The Strategies subscale showed significant positive correlations with the total DIB-R score (r = 0.344; *p* < 0.05) and the interpersonal dimension (r = 0.342; *p* < 0.05), as well as with depressive symptoms (BDI-II, r = 0.502; *p* < 0.05) and trait anxiety (STAI-T, r = 0.521; *p* < 0.05). These findings align with Azad et al. (2025), who reported similar associations between ineffective regulation strategies and heightened psychological distress [60]. Likewise, Ibraheim et al. (2017) highlighted that adolescents with borderline traits display a reduced capacity to mobilize adaptive regulation strategies, which may contribute to both emotional instability and persistent interpersonal difficulties [4]. Such deficits can amplify affective burden and exacerbate relational dysfunctions that are central to BPD.

The Awareness subscale was negatively correlated with the cognitive dimension of the DIB-R (r = −0.404; *p* < 0.05), indicating that individuals who experience greater difficulty identifying their emotions tend to exhibit fewer cognitive distortions as defined by the DIB-R, such as dichotomous thinking, ideas of reference, or projective distortions. This counterintuitive relationship contrasts with Sharp (2015), who proposed that deficits in emotional awareness compromise mentalization capacity and promote hypermentalization, leading to biased interpretations of others’ mental states [9]. Similarly, Azad et al. (2025) found that low emotional awareness is associated with uncertain or distorted mentalization, reducing the accuracy of social judgment and cognitive coherence [60]. In our sample, this pattern may reflect a specific form of cognitive or social disorganization distinctive of borderline functioning [60].

Finally, the total DERS score was positively correlated with depression (r = 0.354; *p* < 0.05), trait anxiety (r = 0.438; *p* < 0.05), and the total DIB-R score (r = 0.309; *p* < 0.05), highlighting the transdiagnostic role of global emotional dysregulation across mood, anxiety, and borderline symptom domains. These correlations converge with Gratz and Roemer (2004), who conceptualized emotional dysregulation as a multidimensional process encompassing deficits in identification, understanding, and modulation of emotions, thereby contributing to both affective distress and impulsive behaviors [23]. They also align with findings from Daros and Williams (2019) and Waite et al. (2025), which identify emotional dysregulation as a core transdiagnostic factor linking anxiety–depression spectra and borderline psychopathology through shared mechanisms of affective disinhibition and impaired cognitive control [27,30].

In this perspective, the global DERS score reflects not only the severity of borderline functioning, but also a shared emotional vulnerability underlying both internalizing disorders and impulsive behaviors, supporting the notion of an emotional–behavioral continuum characteristic of young adults with BPD.

Taken together, these correlations support the hypothesis of a tight interconnection between emotional dysregulation and BPD symptom severity, confirming that the DERS captures not only emotional reactivity but also the interpersonal and cognitive components of borderline psychopathology.

#### 4.3.2. Non-Significant Correlations and Methodological Considerations

Contrary to expectations, no significant correlation was found between the Impulse subscale of the DERS and the Impulsivity dimension of the DIB-R. This finding may appear counterintuitive given the conceptual overlap between these two measures, both of which assess difficulties in inhibiting behavioral responses under conditions of intense emotional arousal. However, several methodological factors may account for this lack of association.

First, the restricted variance within the BPD group (n = 44)—composed exclusively of participants with a confirmed borderline diagnosis—may have limited the detection of significant correlations. The uniformly high levels of DIB-R and DERS scores likely reduced score dispersion, thereby attenuating statistical associations even when they may exist clinically. In a more heterogeneous sample including a broader range of symptom severity, the relationship between emotional impulsivity and behavioral impulsivity would likely have been more pronounced.

Second, although both instruments assess related constructs, they capture different levels of impulsivity. The DERS Impulse subscale reflects the subjective and context-dependent experience of loss of control (state impulsivity), whereas the DIB-R Impulsivity dimension is based on clinician-rated observable behaviors. This dissociation between emotional experience and behavioral manifestation may explain the absence of correlation, suggesting a functional complementarity rather than a contradiction between these two facets of impulsivity.

### 4.4. Correlations Between Emotional Dysregulation, Suicidal Behaviors, and Substance Use

Among the DERS dimensions, the Clarity subscale was negatively correlated with the number of suicide attempts (r = −0.386; *p* < 0.05). Although unexpected, this result suggests that individuals reporting higher levels of emotional confusion in our sample paradoxically reported fewer suicidal behaviors. This counterintuitive association raises several clinical hypotheses. From a developmental perspective, emotional clarity can be conceptualized as a late-emerging emotional competence that relies on the progressive integration of affective experience and higher-order cognitive processes. Longitudinal data indicate that emotional clarity follows a non-linear developmental trajectory during adolescence, with substantial interindividual variability and, in some cases, transient decreases before later consolidation [61]. Neurodevelopmental models further suggest that this vulnerability is underpinned by the protracted maturation of prefrontal regions involved in emotional awareness, reflection, and cognitive control, which continue to develop into early adulthood, whereas limbic systems related to emotional reactivity mature earlier [62] (Ahmed et al., 2015). This fronto-limbic asynchrony may limit the capacity to translate emotional distress into organized, goal-directed behaviors during periods of high emotional load. In this context, low emotional clarity in emerging adults may be associated with confusion, disengagement, or behavioral inhibition rather than impulsive suicidal acting-out. Importantly, this developmental interpretation does not imply that emotional confusion is protective per se, but rather that the relationship between emotional clarity and suicidal behavior may vary according to developmental stage, cognitive maturation, and clinical context, potentially accounting for discrepancies between findings observed in emerging adult samples and those reported in older or more clinically stabilized populations.

A second explanatory hypothesis concerns the role of dissociation as a mechanism of decoupling between subjective emotional experience and behavioral expression of distress. Converging evidence indicates that dissociative experiences are associated with reduced emotional awareness, difficulties identifying and differentiating affects, and a fragmented or disconnected emotional experience. In BPD, dissociation is frequent, particularly under conditions of stress or trauma reactivation, and is associated with altered access to internal emotional states, which may mechanically lead to lower self-reported emotional clarity without implying reduced psychological distress [63].

Beyond impaired emotional awareness, several developmental and biosocial models of BPD describe dissociation as part of multifinal trajectories, including more inhibited or internalizing profiles distinct from impulsive-externalizing patterns. In these trajectories, dissociation may function as a mechanism of emotional and behavioral disengagement, reducing agency, impairing action organization, and inhibiting goal-directed behaviors, including organized self-harm or suicidal acts [64,65]. Empirical studies further suggest that the association between dissociation and suicidality is neither linear nor uniform. While dissociative symptoms may be elevated among individuals with a history of suicide attempts, this relationship appears modest and attenuates when borderline and post-traumatic symptoms are accounted for [66]. Other work indicates that dissociative states characterized by withdrawal, depersonalization, or emotional detachment may be associated with behavioral inhibition and reduced enactment of self-directed behaviors, highlighting the heterogeneity of dissociative–suicidal trajectories [67].

Within this framework, dissociation may act, in a subset of clinically followed young adults, as a decoupling mechanism between subjective emotional confusion and suicidal behavior. Low self-reported emotional clarity may thus reflect a fragmented or poorly accessible emotional experience without translating into increased suicidal behavior. This configuration does not imply lower psychopathological severity but rather points to specific trajectories marked by behavioral inhibition or emotional disengagement.

A further, non-exclusive explanation relates to a temporal misalignment between the variables assessed. Emotional clarity, as measured by the DERS, reflects current subjective emotional functioning, whereas the number of suicide attempts constitutes a cumulative lifetime indicator that may capture behaviors occurring years before study inclusion, potentially during different developmental phases or prior to engagement in specialized care. Thus, low emotional clarity at the time of assessment may coexist with a recent reduction in suicide attempts without reflecting emotional clarity at the time of prior suicidal acts. This temporal discordance represents a classical methodological issue in cross-sectional designs and, in itself, may account for the observed inverse association [68].

An alternative hypothesis is that this inverse association reflects an effect of care context. Patients presenting marked emotional confusion may be identified earlier as clinically vulnerable and benefit from reinforced clinical containment (hospitalization, close ambulatory follow-up, crisis planning), which may reduce the frequency of suicidal behaviors without parallel improvement in subjective emotional clarity. This interpretation remains speculative, as treatment intensity was not quantified in the present study, but is consistent with longitudinal evidence showing that suicidal behaviors may decrease over time under sustained clinical care while subjective vulnerabilities persist [68].

This pattern may also reflect a more inhibited psychological profile, characterized by reduced emotional verbalization or alexithymic traits, in which emotional distress remains poorly articulated at the subjective level while overt self-aggressive behaviors are relatively constrained. Meta-analytic evidence indicates that reduced emotional awareness and alexithymic features are moderately associated with BPD and may bias the subjective reporting of emotional distress, particularly when assessed through self-report measures [57]. Importantly, this configuration should not be interpreted as reflecting lower clinical severity, but rather as a distinct mode of emotional processing and behavioral regulation.

This finding contrasts with a substantial body of empirical evidence underscoring the central role of emotional dysregulation in suicidal behavior. In a longitudinal study, Fortaner-Uyà et al. (2025) demonstrated that the DERS is a robust predictor of suicide attempts among individuals with BPD, particularly through components related to emotional confusion and maladaptive strategies [7]. Similarly, Wyrzykowski et al. (2025) found that low emotional clarity, when combined with high impulsivity, is associated with increased risk-taking behaviors and heightened psychological pain [58]. Moreover, Paris (2005) emphasized the importance of metacognitive processes in suicide prevention, arguing that an inability to identify and differentiate one’s emotions may foster a state of silent affective urgency, which is difficult to mentalize and thus potentially dangerous [69].

Rather than contradicting these models, the present findings highlight the heterogeneity of emotional and behavioral profiles within BPD and underscore the need to consider developmental stage, dissociative processes, care context, and temporal dynamics when interpreting associations between emotional dysregulation and suicidal behavior.

Although our findings do not directly follow this pattern, they highlight the heterogeneity of emotional profiles within BPD and call for a nuanced understanding of the relationship between emotional confusion and suicidality. This suggests the need to explore moderating variables or differentiated developmental trajectories that may influence how emotional confusion manifests in self-destructive behaviors.

In our cohort, the lack of significant associations between DERS scores and substance use indicates that, among young adults with BPD, impulsive behaviors do not necessarily stem from subjectively perceived emotional dysregulation. This dissociation suggests that certain risk behaviors, such as substance use, may not always function as conscious emotional responses, but rather as affective avoidance strategies, sensation-seeking behaviors, or expressions of automatic impulsive processes. According to Billieux et al. (2010), impulsivity is a multidimensional construct, encompassing emotional urgency, stimulation seeking, and low premeditation, which operate partly independently of classical introspective processes [44].

From this perspective, it becomes crucial to distinguish conscious emotional dysregulation—captured by self-report tools such as the DERS—from non-verbalized behavioral impulsivity, which may involve distinct neurocognitive circuits, including those related to motor inhibition and reward sensitivity. In line with this, Mattingley et al. (2024) [70] emphasized that the relationship between emotional impulsivity and addictive behaviors varies depending on levels of distress tolerance and cognitive control. They advocate for multimodal assessment approaches, combining self-report measures, objective behavioral tasks, and neurobiological indicators, to better capture the trajectories leading to risk behaviors [70].

Overall, these findings call for caution in interpreting linear associations between self-reported emotional dysregulation and externalized behaviors. They reinforce the notion that a subgroup of young adults with BPD may exhibit impulsivity not mediated by emotional awareness, presenting specific clinical challenges for detection and intervention. This pattern underscores the need to refine the conceptual link between emotional confusion and suicidal behavior by considering the potential influence of compensatory mechanisms or inhibited profiles that modulate the behavioral expression of emotional distress.

Taken together, these hypotheses suggest that the observed inverse association between emotional clarity and suicide attempts does not contradict existing models of suicidal risk in BPD, but rather reflects developmental, contextual, and methodological factors specific to emerging adulthood.

### 4.5. Relationship Between Emotional Dysregulation and Impulsivity

The correlational analyses conducted in our sample confirm a close functional overlap between impulsivity and emotional dysregulation. The Impulse subscale of the DERS was positively correlated with both Negative Urgency (r = 0.544; *p* < 0.05) and Positive Urgency (r = 0.345; *p* < 0.05), indicating that difficulties in inhibiting behavior under emotional activation are particularly linked to heightened reactivity to intense affective states—whether aversive or euphoric. The total DERS score was also associated with several impulsivity traits—Negative Urgency (r = 0.422), Positive Urgency (r = 0.380), and Lack of Premeditation (r = 0.423)—highlighting a strong interdependence between global emotional dysregulation and reactive impulsivity, especially in contexts of high emotional arousal.

The Strategies subscale of the DERS was significantly correlated with Positive Urgency (r = 0.334), suggesting that limited access to adaptive regulation strategies may facilitate disinhibited behavior under the influence of positive emotions. These findings are consistent with Waite et al. (2025) [30], who demonstrated that positive emotional dysregulation—defined as difficulty modulating intense pleasant affects such as excitement or euphoria—specifically contributes to impulsivity in response to positive emotions, whereas negative emotional dysregulation is more closely linked to disinhibition under distress. These authors emphasize that positive emotional contexts, much like negative ones, can trigger impulsive behaviors in individuals with BPD due to insufficient cognitive control in the face of affective activation [30].

Furthermore, low emotional awareness (Awareness) was correlated with Lack of Premeditation (r = 0.376) and Lack of Perseverance (r = 0.423), indicating that borderline impulsivity also includes attentional and motivational components, reflecting difficulties in maintaining goal-directed control in emotionally charged situations. These results are in line with the model of Weiss, Sullivan, and Tull (2015), who conceptualize risk-taking behaviors as contextual emotion regulation attempts [28]. According to their framework, the inability to accept or modulate emotions—combined with heightened affective reactivity—leads to transient behavioral disinhibition under both negative and positive emotional states [28].

Additionally, findings from Waite et al. (2025) reinforce this interactional dynamic: in a clinical cohort, the combination of high emotional urgency and emotion dysregulation (both negative and positive) was significantly associated with self-damaging behaviors, including self-injury and risky sexual behaviors [30]. These authors highlight the predictive value of this combined profile—emotional impulsivity coupled with ineffective regulation—in explaining borderline functioning [30].

Complementarily, Sebastian et al. (2013) proposed a model in which the synergy between contextual impulsivity and emotional dysregulation constitutes a major vulnerability factor, particularly when individuals lack adaptive affective modulation strategies [25]. In a similar vein, Mattingley et al. (2024) [70] confirmed that the relationship between emotional impulsivity and risk behaviors varies according to levels of distress tolerance and cognitive control, advocating for an integrative assessment approach that combines subjective evaluation, behavioral indicators, and neurocognitive markers to better understand the dynamics underlying impulsive and self-destructive actions [61].

#### 4.5.1. Toward an Integrated Model of Emotional Dysregulation and Impulsivity in Borderline Personality Disorder

The joint analysis of DERS and UPPS-P scores in our sample reinforces previous findings by revealing robust associations between emotional dysregulation and impulsivity traits. Emotional impulsivity was strongly related to both Negative Urgency and Positive Urgency, suggesting that young adults with BPD react impulsively not only to distressing emotions but also to intensely pleasant affects. This bifaceted pattern of emotional impulsivity is characteristic of borderline functioning, as shown by Mungo et al. (2025), who identified these urgency dimensions as specific markers of the disorder [37].

Moreover, the observed associations between low emotional awareness and traits such as Lack of Premeditation and Lack of Perseverance indicate that borderline impulsivity cannot be reduced to mere emotional reactivity; it also involves executive dysfunctions in planning and goal maintenance. These findings support the idea that impulsive behaviors in BPD reflect a dual deficit, encompassing both affective and cognitive regulatory systems.

The study by Iverson et al. (2012) provides complementary insight into the link between emotion regulation and impulsivity in BPD [71]. The authors demonstrated that experiential avoidance—the tendency to escape or suppress negative emotions rather than tolerate them—is the main determinant of BPD symptom severity in young adults [71]. This mechanism can be conceptualized as a form of emotional impulsivity, wherein action serves as a means of alleviating acute affective overload. These findings reinforce the biosocial model, which conceptualizes dysfunctional behaviors as compensatory emotion regulation strategies developed in response to invalidating environments [12].

Taken together, these results highlight the functional interdependence between emotional systems and impulsivity dimensions in young adults with BPD. They support the view that emotional dysregulation and emotional impulsivity represent two interwoven facets of a single process of affective disinhibition, which emerges early and becomes fully expressed during the transition to adulthood. This developmental profile may explain the high frequency of risk-taking and self-damaging behaviors in this population and underscores the need to jointly target these dimensions in therapeutic interventions.

In this context, mindfulness-based, distress-tolerance, and emotion-regulation–focused approaches, such as those integrated within dialectical behavior therapy (DBT) and contemporary transdiagnostic treatments, appear particularly well suited to reducing impulsive reactivity and enhancing emotional mastery in young adults with BPD.

#### 4.5.2. Interaction Between Trait and State Impulsivity

The distinction between trait impulsivity and state impulsivity is crucial for refining the understanding of risk behaviors among young adults with BPD. On one hand, the UPPS-P scale assesses trait-dependent dimensions of impulsivity—stable tendencies to act in a disinhibited manner across contexts, regardless of the immediate emotional state [44]. On the other hand, specific components of the DERS, particularly the Impulse subscale, capture context-dependent impulsivity, which is reactive to emotional distress. Gratz and Roemer (2004) emphasized that this difficulty in inhibiting impulsive behaviors occurs primarily “in response to emotional distress, rather than as a general characteristic of the individual” [23]. In other words, the DERS reflects manifestations of state impulsivity, rooted in momentary affective fluctuations.

This complementarity between trait and state impulsivity is particularly relevant given that both forms appear to interact in the emergence of problematic behaviors. Recent integrative models suggest that dispositional traits—such as emotional urgency—predispose individuals to impulsive responses, but that their expression is modulated by the intensity of emotional states and by the available regulatory capacities [37,70]. Accordingly, the functional overlap observed between DERS and UPPS-P scores in our sample underscores the importance of jointly considering acute emotional reactivity and dispositional vulnerabilities when conceptualizing risk-taking behaviors in BPD.

Taken together, these findings emphasize that understanding impulsivity in BPD requires a multidimensional perspective that accounts for both trait-like predispositions and state-dependent fluctuations in emotional control.

However, the cross-sectional nature of our design does not allow for determining the directionality of the observed relationships between emotional dysregulation and impulsivity. Moreover, the lack of behavioral or neurocognitive measures limits the explanatory power of our results, which should be further explored through multimodal and longitudinal methodologies.

Overall, our findings underscore a dynamic synergy between emotional dysregulation and impulsivity, suggesting that these constructs are not independent entities but rather interdependent expressions of a shared affective disinhibition process, modulated by emotional reactivity and cognitive control.

This interactional profile, particularly salient during the transition to adulthood, aligns with transactional and biosocial models [9,11], in which constitutional emotional vulnerability, when combined with an invalidating environment, promotes the emergence of a deficient regulatory style and reactive impulsivity.

### 4.6. Strengths and Limitations of the Study

Despite the overall consistency of the findings and the robustness of several observed associations, a number of methodological strengths and limitations must be discussed to better contextualize the scope of the conclusions and to inform future research directions.

The clinical context in which this study was conducted represents a key interpretative element. The specificity of the recruitment process constitutes a major methodological strength: participants were included in a real-world psychiatric care setting, without exclusions related to symptom severity or comorbidity complexity. This choice enhances the ecological validity of the results, allowing them to more accurately reflect the clinical reality of young adults treated for BPD—typically characterized by high symptomatic heterogeneity and complex care trajectories.

One of the main strengths of this study also lies in its developmental anchoring, focusing on the critical transition to adulthood (ages 16–25)—a period of heightened vulnerability to emotional dysregulation and impulsive behaviors. This developmental perspective improves understanding of the early emergence of borderline features and helps identify at-risk profiles at a key stage for prevention and early intervention.

The study further distinguishes itself by its multidimensional and integrative design, combining measures of emotion regulation (DERS), impulsivity (UPPS-P), and clinical variables from the DIB-R, BDI-II, and STAI-T. This design allowed for a nuanced assessment of the underlying psychological mechanisms. The use of adjusted statistical models controlling for major clinical covariates (anxiety, depression, psychiatric history) reinforced the specificity of the findings, particularly concerning emotional impulsivity and deficits in emotional awareness and clarity as robust discriminative markers of BPD.

Another methodological strength is the rigorous control of depressive symptoms, frequently associated with BPD but potentially confounding the assessment of emotional regulation. By adjusting analyses for depressive symptomatology, we isolated emotion-specific mechanisms intrinsic to BPD, independent of generalized negative affectivity. This distinction strengthens the internal validity of our results and delineates BPD-specific emotional dysfunctions from transdiagnostic depressive processes.

Findings from Dixon-Gordon et al. (2015) support this differentiation: although depressive symptomatology is linked to elevated negative affectivity, it does not entail the same emotional disturbances observed in BPD [72]. The authors showed that prolonged emotional reactivity, difficulty inhibiting impulsive behaviors under distress, and severe emotion regulation impairments are specific to BPD and not to depression alone. Their comparative analyses across three groups—BPD, MDD, and comorbid BPD–MDD—revealed that borderline traits uniquely predicted increased emotional intensity, persistent fear-related anxiety, and impaired behavioral control. Conversely, comorbidity between BPD and MDD amplified these disturbances, yielding a synergistic emotional dysregulation profile [72].

Taken together, these results reinforce the validity of our analytic approach and confirm that BPD represents a distinct emotional phenotype, marked by intense, enduring, and poorly regulated affectivity closely linked to distress-related impulsivity. The study also highlights functionally meaningful correlations between emotional dysregulation, impulsivity, and symptom severity, in line with biosocial and developmental models of BPD.

However, several methodological limitations should be noted. The modest sample size, particularly in the BPD group, limited statistical power and increased the risk of Type II error, reducing the ability to detect small to moderate effects or interaction terms (e.g., gender or comorbidities).

The gender imbalance, with a strong predominance of female participants (95% in the BPD group), restricts the generalizability of findings to males. However, this distribution reflects clinical recruitment in adolescent and emerging adult psychiatry, where young women are more frequently identified, referred, and hospitalized for BPD during this developmental period. Given that certain facets of emotion regulation and impulsivity are known to vary by sex, this aspect deserves further exploration in gender-stratified studies.

It is also important to note that some theoretically expected associations between DERS subscales and clinical variables did not reach significance. This does not undermine the theoretical framework but likely reflects the heterogeneity of emotional dysregulation profiles among young adults with BPD. Prior studies suggest that dysregulation can manifest as either hyperactivated (emotional reactivity, impulsivity) or deactivated (emotional numbing, inhibition), depending on attachment style and coping strategies [8]. Such interindividual variability may attenuate statistical correlations, underscoring the need for typological or differential approaches to fully capture the emotional complexity of BPD.

Although ADHD comorbidity is frequently cited as a factor that exacerbates emotional dysregulation, it was underrepresented in our sample (n = 5), preventing a robust analysis of its contribution. Nonetheless, studies have shown that BPD–ADHD comorbidity is associated with higher emotional instability and impulsivity than either condition alone [73,74]. These findings are reported here to contextualize existing literature and should not be interpreted as evidence derived from the present sample.

Beyond ADHD, the absence of a clinical comparison group (e.g., MDD, ADHD-only, PTSD) constitutes a key limitation, as it precludes determining whether the observed emotional alterations are specific to BPD or shared across disorders characterized by affective instability. This design choice was intentional, as the primary objective of the study was to contrast emerging adults with a clinically confirmed diagnosis of BPD with a non-clinical comparison group, rather than to disentangle disorder-specific versus transdiagnostic mechanisms. Future research should therefore include differentiated clinical groups and employ multimodal approaches (subjective, behavioral, and neurobiological) to clarify the emotional specificities of BPD.

Suicidal behaviors were assessed using clinician-rated information from the Diagnostic Interview for Borderlines–Revised (DIB-R); however, only the number of lifetime suicide attempts was extracted and analyzed, and other dimensions of suicidality (e.g., suicidal ideation intensity or non-suicidal self-injury) were not systematically assessed.

However, these results should be interpreted with caution. Although the regression analyses followed established methodological standards—including an adequate subject-to-variable ratio (≥10 participants per covariate), the use of robust standard errors, and verification of model quality (Hosmer–Lemeshow and Link tests)—the relatively small sample size may have broadened confidence intervals and reduced the precision of effect estimates. Moreover, the lack of significant associations for some DERS subdimensions should not be interpreted as evidence of a true absence of effect. Given the limited statistical power, the study was likely underpowered to detect small to moderate effects, thus increasing the risk of Type II error. In this context, non-significant results may reflect insufficient power rather than a genuine absence of relationships between certain facets of emotion regulation and BPD features.

Finally, the cross-sectional design precludes causal inference. It remains impossible to determine whether emotional dysregulation precedes BPD symptomatology or emerges as a consequence of it. Longitudinal studies involving larger and clinically balanced samples will be necessary to confirm the stability and specificity of these associations over time.

The exclusive reliance on self-report measures (DERS, UPPS-P, STAI-T, BDI-II) introduces potential biases of introspection and social desirability, particularly among individuals with unstable emotional self-awareness. This limitation is particularly relevant in BPD, as meta-analytic evidence indicates that reduced emotional awareness and alexithymic traits are moderately associated with borderline pathology, while also highlighting that the predominant reliance on self-report measures may bias the assessment of emotional processes [57]. Moreover, the absence of behavioral and neurocognitive measures limits construct validity, as some overlap between DERS (Impulse, Goals) and UPPS-P (Urgency, Lack of Premeditation) may reflect conceptual redundancy. Future studies should integrate objective measures (behavioral tasks, physiological markers, neural connectivity indices) to better differentiate state vs. trait impulsivity and to refine the clinical interpretation of these constructs. Future studies would benefit from combining trait-based measures such as the DERS with state-level assessments (e.g., ecological momentary assessment or experimental paradigms) to better capture dynamic emotion regulation processes.

Lastly, self-selection bias cannot be ruled out: participants more aware of or concerned by their emotional difficulties may have been more inclined to participate, thereby limiting the representativeness of the sample.

### 4.7. Future Directions

The findings of this study open several promising research avenues aimed at deepening the understanding of the emotional and impulsive mechanisms involved in BPD during the transition to adulthood.

#### 4.7.1. Longitudinal Approaches and Developmental Trajectories

A first perspective involves adopting a longitudinal approach to examine the evolution of emotion regulation components over time. The cross-sectional data from the present study suggest that certain dimensions—particularly emotional awareness and clarity—may constitute early markers of borderline functioning. It would therefore be relevant to track these dimensions over several years to determine their predictive role in symptom stabilization or remission. Such follow-up studies could provide a clearer understanding of the developmental trajectories of BPD and help identify protective factors that promote emotional recovery and adaptive functioning in adulthood.

#### 4.7.2. Exploring Neurocognitive and Biological Mediators

The psychometric results and the correlations observed between the DERS, DIB-R, and UPPS-P highlight the need for multimodal approaches that integrate behavioral, neurocognitive, and biological measures.

Recent evidence from neuroimaging and electrophysiological studies confirms that emotional dysregulation in BPD is associated with specific neural abnormalities affecting fronto-limbic circuits involved in emotion processing and regulation. Göhre et al. (2025) reported that patients with BPD or post-traumatic stress disorder (PTSD) display reduced P3 amplitudes and altered late positive potentials (LPP) in response to emotional stimuli, reflecting impaired cortical processing of emotions [22]. These electrophysiological alterations are interpreted as markers of limbic hyperactivation coupled with deficient prefrontal control, consistent with Linehan’s biosocial model [22].

These findings suggest that neurophysiological abnormalities are not confined to acute stress or trauma-related contexts but rather reflect a transversal vulnerability to emotional reactivity. In this perspective, future studies would benefit from incorporating multimodal measures—including EEG, functional neuroimaging, and physiological indicators of reactivity (e.g., heart rate, skin conductance)—in parallel with self-reported measures of emotion regulation. Such designs would allow examination of the correspondence between behavioral, cognitive, and neural dimensions of emotional regulation and the identification of integrative biomarkers of borderline functioning.

#### 4.7.3. Experimental and Ecological Assessment of Emotion Regulation

Future research could also rely on experimental paradigms targeting emotional reactivity and real-time regulation strategies, particularly through reappraisal, inhibition, or distress tolerance tasks.

The study by Göhre et al. (2025) showed that, despite reported difficulties in spontaneously using adaptive strategies, patients with BPD or PTSD were able to apply cognitive reappraisal effectively when properly trained [22]. This observation suggests that emotional dysregulation does not stem from an irreversible structural deficit but rather from a limited and inflexible access to adaptive strategies, reinforcing the relevance of experimental studies focused on learning and applying emotion regulation skills [22].

In parallel, experience sampling methods (ESM) represent a promising tool to evaluate affective variability in ecological settings. Using ESM, Moukhtarian et al. (2021) demonstrated that adults with BPD and those with ADHD show comparable levels of emotional intensity and instability in daily life, although their triggers differ—with borderline fluctuations being more closely related to interpersonal situations [21].

These findings suggest that combining laboratory-based paradigms (assessing explicit regulation capacities) with ESM-based measures (capturing real-life emotional dynamics) could yield a more precise understanding of borderline emotional functioning and its contextual determinants. A multimodal approach of this kind would overcome the limitations of static evaluations by capturing emotion regulation in its temporal, situational, and neurobiological dimensions.

#### 4.7.4. Therapeutic Targets and Early Prevention

The findings of this study suggest that specific dimensions of emotion regulation—particularly emotional impulsivity, awareness, and clarity—represent key intervention targets for the prevention and early treatment of BPD. These components correspond to those identified by Salgó et al. (2021), who reported that individuals with BPD exhibit significant deficits in mindfulness, emotional clarity, and self-compassion, indicating that these abilities should be specifically trained to restore emotional flexibility [55].

Schmidt et al. (2024) confirmed that mindfulness-based modules progressively reduce impulsive reactions and emotional distress by enhancing non-judgmental awareness, bodily attention, and decentering capacities [24]. These findings suggest that restoring a more tolerant and reflective relationship with one’s emotional experience constitutes a central therapeutic pathway for preventing symptom chronicity in BPD [24].

Dialectical Behavior Therapy (DBT) has demonstrated strong efficacy in reducing emotional dysregulation and self-damaging behaviors, including in emerging or subthreshold forms of BPD. Beyond global efficacy, several studies have revealed specific modulation of distinct DERS components following DBT interventions. Gratz et al. (2015) showed that, among women with BPD and self-injurious behaviors, the most substantial improvements occurred in the Nonacceptance and Impulse dimensions, and that these changes mediated reductions in affective and cognitive symptoms [75]. Similarly, Neacsiu et al. (2014) demonstrated, in a transdiagnostic randomized trial, that DBT skills training significantly reduced total DERS scores—particularly Nonacceptance, Impulse, and Strategies—through the acquisition and application of behavioral skills [76].

Furthermore, Sharp et al. (2011) observed in adolescents with borderline traits that deficits in emotional awareness and clarity (DERS Awareness and Clarity subscales) mediated the link between hypermentalization and borderline symptom severity, suggesting an overlap with Mentalization-Based Therapy (MBT) processes [77]. In a complementary framework, Sharp (2015) noted that BPD is often characterized by hypermentalization, i.e., an excessive and inaccurate interpretation of others’ mental states, which contributes to interpersonal instability [9]. Integrating mentalization-focused modules (such as MBT) may therefore enhance both affective regulation and relational stability at early stages of intervention.

A differentiated analysis of emotion regulation subdimensions can thus guide tailored therapeutic strategies: enhancing acceptance and distress tolerance (DBT), improving emotional understanding and differentiation (MBT), or strengthening identity coherence and goal-directed control (psychodynamic therapies such as TFP).

From a developmental perspective, several recent studies underscore the importance of early and stepped interventions in BPD prevention. The longitudinal study by Cavelti et al. (2024) illustrates the effectiveness of a stepped-care model for adolescents with emerging BPD symptoms or self-harming behaviors [36]. In this model, all participants first received a brief, low-intensity intervention focused on self-harm reduction (Cutting Down Program—CDP), while only those with persistent symptoms (≥3 BPD criteria and ZAN-BPD ≥6) were referred to a more intensive therapy (DBT-A) [36].

Results indicated that the CDP alone led to sustained improvement in psychosocial functioning and a reduction in BPD criteria over two years, demonstrating the feasibility and cost-effectiveness of a stepped-care framework that allocates intensive resources only to persistent or severe cases. The authors highlight that such a model—by ensuring rapid access to emotion regulation interventions—may prevent the consolidation of dysfunctional patterns characteristic of BPD [36].

This logic of graduated intervention aligns with Chanen et al. (2017), who advocate for viewing BPD prevention as a public health priority, justifying the systematic integration of early identification and treatment into adolescent and young adult mental health services [10].

Finally, recent studies such as Azad et al. (2025) highlight the role of deficits in mentalization and low tolerance of emotional uncertainty in the severity of addictive behaviors among young adults, supporting the need for transdiagnostic programs targeting emotion regulation, mindfulness, and mentalization simultaneously [60].

Overall, future prevention and treatment protocols for BPD should rely on integrated and adaptive models, combining emotion regulation skills training, mindfulness- and mentalization-based interventions, and stepped-care approaches tailored to both severity level and developmental stage. Such early, flexible, and multimodal strategies could reduce impulsive symptomatology, enhance distress tolerance, and foster long-term emotional resilience in vulnerable young adults.

## 5. Conclusions

The present findings confirm the central role of emotional dysregulation in borderline personality disorder (BPD) during the transition to adulthood and underscore its discriminative value relative to other forms of psychopathology. Among the DERS subdimensions, difficulties in controlling emotional impulses, low emotional awareness, and poor emotional clarity emerged as independent predictors of BPD diagnosis, even after controlling for depressive and anxiety symptoms. These components reflect a pattern of behavioral and cognitive dysregulation, consistent with Linehan’s (1993) [11] biosocial model and its recent empirical validation in adolescents [15], in which emotional vulnerability, impulsivity, and parental invalidation interact through a transactional process that maintains borderline symptomatology [11].

This pattern aligns with the Gratz and Roemer (2004) model, which conceptualizes emotion regulation as comprising interdependent components of identification, understanding, and modulation [23]. Our results converge with those of Rufino et al. (2017), who identified a Global Dysregulation subtype among BPD patients characterized by reduced clarity and increased impulsivity [56]. Complementarily, Waite et al. (2024) demonstrated that negative and positive emotional dysregulation contribute differently to behavioral impulsivity—the former being associated with disinhibition under distress, and the latter with sensation-seeking [30].

Taken together, the Impulse–Awareness–Clarity triad identified in this study appears to represent a discriminant marker of emerging BPD. The inability to identify and understand one’s emotions, combined with heightened emotional impulsivity, seems to foster behavioral disinhibition and interpersonal distress. These mechanisms can be interpreted through mentalization-based frameworks, which describe the temporary collapse of reflective functioning under intense emotional arousal. Hence, mentalization deficits and emotional dysregulation can be viewed as two complementary expressions of the same affective-cognitive disorganization process.

From a clinical perspective, these findings highlight the importance of early, developmentally tailored, and stepped-care interventions targeting emotion regulation and mentalization as soon as early signs of vulnerability appear. Following the recommendations of Bozzatello et al. (2021), structured identification of young individuals showing high emotional reactivity and regulatory difficulties may facilitate early referral to specialized programs [19]. Evidence-based interventions derived from Dialectical Behavior Therapy (DBT) and Mentalization-Based Therapy (MBT), specifically adapted for individuals aged 16–25 years, appear particularly relevant within this framework [19]. By fostering emotional clarity, distress tolerance, and reflective awareness of mental states, such interventions may promote affective stabilization, impulsivity control, and long-term emotional resilience in young adults with emerging BPD.

## Figures and Tables

**Table 1:** Sociodemographic and General Clinical Characteristics of Participants (*n* = 184).

Variables	Categories	%	Non-Clinical Group (*n* = 140)	BPD Individuals (*n* = 44)	*p*-Value Chi^2^
Gender	Male (*n* = 39)	21.2%	26.4%	4.6%	0.002
	Female (*n* = 145)	78.8%	73.6%	95.4%	
Student	No (*n* = 10)	5.4%	5.0%	6.8%	0.643
	Yes (*n* = 174)	94.6%	95.0%	93.2%	
Somatic and/or surgical history	No (*n* = 80)	43.5%	44.3%	40.9%	0.693
	Yes (*n* = 104)	56.5%	55.7%	59.1%	
Psychiatric history	No (*n* = 160)	87.0%	97.1%	54.6%	<0.001
	Yes (*n* = 24)	13.0%	2.9%	45.4%	
Psychiatric comorbidities	0 (*n* = 154)	83.6%	100.0%	31.8%	<0.001
	1 (*n* = 15)	8.2%	0.0%	34.1%	
	≥2 (*n* = 15)	8.2%	0.0%	34.1%	
Alcohol	No (*n* = 54)	29.4%	32.9%	18.2%	0.145
	Occasional (*n* = 104)	56.5%	52.9%	68.2%	
	Binge drinking (*n* = 26)	14.1%	14.2%	13.6%	
Drugs	No (*n* = 157)	85.3%	95.7%	52.3%	<0.001
	Yes (*n* = 27)	14.7%	4.3%	47.7%	
Family psychiatric history	No (*n* = 125)	67.9%	85.7%	11.4%	<0.001
	Yes (*n* = 59)	32.1%	14.3%	88.6%	
History of suicide attempts	No (*n* = 141)	76.6%	93.6%	22.7%	<0.001
	Yes (*n* = 43)	23.4%	6.4%	77.3%	
Past psychological follow-up	No (*n* = 136)	73.9%	90.7%	20.5%	<0.001
	Yes (*n* = 48)	26.1%	9.3%	79.5%	
Actual psychological follow-up	No (*n* = 140)	76.1%	100.0%	0.0%	<0.001
	Yes (*n* = 44)	23.9%	0.0%	100.0%	
Psychotropic treatment	No (*n* = 152)	82.6%	100.0%	27.3%	<0.001
	Yes (*n* = 32)	17.4%	0.0%	72.7%	
Educational level	Primary education (*n* = 113)	61.4%	58.6%	70.5%	0.158
	Secondary or Higher education (*n* = 71)	38.6%	41.4%	29.5%	
	**Median** **(P25–P75)**				***p*-Value Wilcoxon test**
Age (years)	18.0 (17.0–20.0)		18.0 (17.0–20.0)	17.0 (16.0–20.5)	0.056
Number of suicide attempts	0.0 (0.0–0.0)		0.0 (0.0–0.0)	2.0 (1.0–5.0)	<0.001

**Table 2:** Psychometric Characteristics of the Sample [37].

Variables	Median (P25–P75)	Non-Clinical group (*n* = 140)	BPD Individuals (*n* = 44)	*p*-Value Wilcoxon Test
*Emotion dysregulation*				
DERS Non Acceptance	13.0 (9.0–22.0)	12.0 (8.0–18.0)	22.0 (17.0–26.0)	<0.001
DERS Goals	19.0 (14.0–23.0)	17.0 (13.0–21.0)	23.0 (21.0–25.0)	<0.001
DERS Impulse	15.0 (10.0–21.0)	13.0 (9.0–18.0)	24.0 (18.0–29.0)	<0.001
DERS Awareness	17.0 (13.0–21.0)	15.0 (12.0–19.0)	22.0 (18.0–25.0)	<0.001
DERS Clarity	14.0 (9.0–18.0)	12.0 (9.0–17.0)	18.0 (15.0–21.0)	<0.001
DERS Strategies	24.0 (17.0–32.0)	21.0 (14.0–28.0)	33.0 (28.0–37.0)	<0.001
DERS total	104.0 (80.0–129.0)	93.0 (74.0–114.0)	138.0 (125.0–149.0)	<0.001
*Mood and Anxiety symptoms*				
BDI	14.0 (9.0–26.0)	12.0 (8.0–18.0)	33.0 (19.0–45.0)	<0.001
Spielberger–trait	50.0 (39.0–60.0)	46.0 (37.0–53.0)	66.0 (57.0–72.0)	<0.001
*Impulsivity Traits*				
UPPS urgencies negative	11.0 (8.0–13.0)	10.0 (8.0–12.0)	14.0 (11.0–15.0)	<0.001
UPPS urgencies positive	13.0 (10.0–14.0)	12.0 (10.0–14.0)	14.0 (12.0–16.0)	<0.001
UPPS lacks premeditation	9.0 (7.0–11.0)	8.0 (7.0 -10.0)	11.0 (9.0–13.0)	<0.001
UPPS lacks perseverance	8.0 (6.0–10.0)	7.0 (5.0–9.0)	11.0 (9.0–12.0)	<0.001
UPPS seeks sensation	12.0 (9.0–14.0)	11.0 (9.0–14.0)	13.0 (9.0–15.0)	0.119

**Table 3:** Scores on the Diagnostic Interview for Borderlines—Revised (DIB-R) Among Participants with BPD (*n* = 44) [37].

Dimension	Median (P25–P75)
Affective Dimension	9.0 (8.0–10.0)
Cognitive Dimension	3.0 (2.0–4.0)
Impulsive Dimension	7.0 (5.0–8.0)
Interpersonal Dimension	10.0 (8.0–12.0)
DIB-R Total Score	9.0 (8.0–10.0)

**Table 4:** Adjusted results for DERS (*n* = 184).

Variables	b_a1_ (SE) Non-Clinical Group vs. BPD Individuals
DERS Non Acceptance	3.5 (3.4)
DERS Goals	4.0 (2.9)
DERS Impulse	8.1 (3.2) *
DERS Awareness	10.5 (2.8) *
DERS Clarity	6.1 (2.2) *
DERS Strategies	6.0 (3.7)
DERS total	37.4 (11.4) *

b_a1_ (ES): adjusted quantile regression coefficient (standard error). Coefficients represent the adjusted difference in median DERS scores between the non-clinical group and individuals with BPD. Models were adjusted for gender, psychiatric history, psychiatric comorbidities, substance use, family psychiatric history, number of suicide attempts, past and current psychological follow-up, psychotropic treatment, BDI-II score, and STAI-T score.; * *p* < 0.05.

**Table 5:** Multivariate analyses (n = 184).

Variables	%	Non-Clinical Group	BPD Individuals	Model 1 OR Unadjusted (CI 95%)	*p*-Value	Model 2 OR Adjusted (CI 95%)	*p*-Value
DERS Impulse							
≤15 (n = 100)	54.4%	66.4%	15.9%	1	<0.001	1	<0.001
>15 (n = 84)	45.6%	33.6%	84.1%	10.45 (4.34 to 25.23)		5.91 (2.27 to 15.37)	
DERS Awareness							
≤17 (n = 100)	54.4%	63.6%	25.0%	1	<0.001	1	0.003
>17 (n = 84)	45.6%	36.4%	75.0%	5.24 (2.44 to 11.24)		3.56 (1.52 to 8.35)	
DERS Clarity							
≤15 (n = 109)	59.2%	69.3%	27.3%	1	<0.001	1	0.013
>15 (n = 75)	40.8%	30.7%	72.7%	6.01 (2.83 to 12.79)		2.90 (1.25 to 6.73)	
DERS total							
≤129 (n = 139)	75.5%	90.7%	27.3%	1	<0.001	1	0.008
>129 (n = 45)	24.5%	9.3%	72.3%	26.05 (10.86 to 62.50)		12.08 (4.54 to 32.16)	

Model 1 = Model unadjusted. Model 2 = Model adjusted for gender, BDI score and Spielberger–Trait score.

**Table 6:** Correlation between BPD dimensions and DERS dimensions (*n* = 44).

	DIB R Total	Affective Dimension	Cognitive Dimension	Impulsive Dimension	Interpersonal Dimension	BDI Score	Spielberger–Trait Score
DERS Non Acceptance	0.254	0.312 *	0.240	0.085	0.201	0.013	0.114
DERS Goals	−0.017	0.064	−0.188	0.009	0.062	0.350 *	0.301 *
DERS Impulse	0.317 *	0.246	0.147	0.258	0.290	0.155	0.244
DERS Awareness	−0.026	−0.125	**−0.404** *	−0.066	−0.240	0.164	0.211
DERS Clarity	−0.068	0.121	−0.116	−0.166	−0.114	0.171	0.233
DERS Strategies	0.344 *	0.141	0.171	0.156	0.342 *	**0.502** *	**0.521** *
DERS Total	0.309 *	0.237	0.004	0.164	0.217	0.354 *	**0.438** *

* *p* < 0.05; Correlations with an absolute value ≥ 0.40 (|r| ≥ 0.40), whether positive or negative, are considered of moderate to large magnitude and are highlighted to facilitate clinical interpretation. Bold formatting is used to highlight correlations with an absolute value ≥ 0.40 (|r| ≥ 0.40), whether positive or negative, as these are considered of moderate to large magnitude and are emphasized to facilitate clinical interpretation.

**Table 7:** Correlation between DERS dimensions and other impulsive markers for BPD (*n* = 44).

	Number of Suicide Attempts	History of Suicide Attempts	Drugs Consumption
DERS Non Acceptance	−0.203	−0.036	0.016
DERS Goals	−0.184	−0.002	0.109
DERS Impulse	0.045	0.066	−0.005
DERS Awareness	−0.113	0.075	0.034
DERS Clarity	−0.386 *	−0.264	−0.140
DERS Strategies	0.034	0.206	0.133
DERS total	−0.153	0.053	0.081

* *p* < 0.05.

**Table 8:** Correlation between DERS dimensions and UPPS dimensions for BPD individuals.

	UPPS Urgencies Negative	UPPS Urgencies Positive	UPPS Lacks Premeditation	UPPS Lacks Perseverance	UPPS Seeks Sensation
DERS Non Acceptance	0.254	0.077	0.032	−0.253	−0.014
DERS Goals	0.275	0.154	0.246	0.192	−0.120
DERS Impulse	**0.544** *	0.345 *	0.286	0.186	−0.107
DERS Awareness	0.180	0.121	0.376 *	**0.423** *	0.074
DERS Clarity	0.067	0.295	0.158	0.020	0.075
DERS Strategies	0.131	0.334 *	0.159	0.035	−0.242
DERS total	**0.422** *	0.380 *	**0.423** *	0.245	−0.126

* *p* < 0.05. Correlations with an absolute value ≥ 0.40 are considered of moderate to large magnitude and are highlighted to facilitate clinical interpretation. Bold formatting is used to highlight correlations with an absolute value ≥ 0.40 (|r| ≥ 0.40), whether positive or negative, as these are considered of moderate to large magnitude and are emphasized to facilitate clinical interpretation.

## Data Availability

The corresponding author may provide the data for this study upon reasonable request.
